# DNA methylation of *Vesicular Glutamate Transporters* in the mesocorticolimbic brain following early-life stress and adult ethanol exposure—an explorative study

**DOI:** 10.1038/s41598-021-94739-8

**Published:** 2021-07-28

**Authors:** Maria Vrettou, Liying Yan, Kent W. Nilsson, Åsa Wallén-Mackenzie, Ingrid Nylander, Erika Comasco

**Affiliations:** 1 Department of Neuroscience, Science for Life Laboratory, Uppsala University, Uppsala, Sweden; 2 EpigenDx, Inc, Hopkinton, MA USA; 3 Centre for Clinical Research Västerås, Uppsala University, Västmanland County Hospital Västerås, Uppsala, Sweden; 4 The School of Health, Care and Social Welfare, Mälardalen University, Västerås, Sweden; 5 Department of Organismal Biology, Uppsala University, Uppsala, Sweden; 6 Department of Pharmaceutical Biosciences, Uppsala University, Uppsala, Sweden

**Keywords:** Genetics, Neuroscience

## Abstract

DNA methylation and gene expression can be altered by early life stress (ELS) and/or ethanol consumption. The present study aimed to investigate whether DNA methylation of the Vesicular Glutamate Transporters (*Vglut*)*1-3* is related to previously observed *Vglut1-3* transcriptional differences in the ventral tegmental area (VTA), nucleus accumbens (Acb), dorsal striatum (dStr) and medial prefrontal cortex (mPFC) of adult rats exposed to ELS, modelled by maternal separation, and voluntary ethanol consumption. Targeted next-generation bisulfite sequencing was performed to identify the methylation levels on 61 5′-cytosine-phosphate-guanosine-3′ sites (CpGs) in potential regulatory regions of *Vglut1*, 53 for *Vglut2*, and 51 for *Vglut3*. In the VTA, ELS in ethanol-drinking rats was associated with *Vglut1-2* CpG-specific hypomethylation, whereas bidirectional *Vglut2* methylation differences at single CpGs were associated with ELS alone. Exposure to both ELS and ethanol, in the Acb, was associated with lower promoter and higher intronic *Vglut3* methylation; and in the dStr, with higher and lower methylation in 26% and 43% of the analyzed *Vglut1* CpGs, respectively. In the mPFC, lower *Vglut2* methylation was observed upon exposure to ELS or ethanol. The present findings suggest *Vglut1-3* CpG-specific methylation signatures of ELS and ethanol drinking, underlying previously reported *Vglut1-3* transcriptional differences in the mesocorticolimbic brain.

## Introduction

Adversity during early life has been linked to psychopathology later in life, including alcohol use disorder (AUD)^1–3^. Notably, early life stress (ELS) can lead to epigenetic modifications, such as alterations in DNA methylation patterns, which can in turn affect gene expression^4^. DNA methylation, catalyzed by DNA (cytosine-5)-methyltransferases (DNMTs), is a chemical modification, suggested to serve as a signature of early life experiences that can imprint on the developing brain, explaining why those early experiences have a long-lasting effect^1^.

In rats, ELS can be modeled via maternal separation (MS) daily for 60 to 360 min, whereas MS for only 15 min (MS15) is more naturalistic, thus serving as control condition^5^. MS360 has been associated with higher alcohol consumption^5^, while MS15 leads to hypo-reactive hypothalamic–pituitary–adrenal (HPA) axis to stress^6^ and has a protective role towards alcohol intake^5^. The effect of MS is mediated via disturbances in mother–pup interactions and maternal care towards the offspring^5,6^. Differences in maternal care in rats and humans have been associated with altered HPA-axis response to stress and negative mental health via differential methylation of the promoter of the glucocorticoid receptors^7,8^.

Not only ELS, but also ethanol consumption per se*,* can affect DNA methylation patterns^9^. Chronic ethanol exposure has been associated with global DNA hyper-methylation in various brain regions of rodents^9^, and specifically associated with increased DNMT1 expression in the nucleus accumbens (Acb) of adult mice^10^ and in the Acb and medial prefronal cortex (mPFC) of post-dependent rats after weeks of abstinence to ethanol^11^. On the contrary, though measured in blood, a recent epigenome-wide association study (EWAS) in humans showed greater alcohol intake to be associated with lower global DNA methylation^12^. Yet, the effects of both ELS and ethanol on DNA methylation in the young adult non-dependent brain are largely unknown. Indeed, to disentangle the biological underpinnings of the initial effects of ethanol use can contribute to shed light on the potential neural mechanisms leading to the development of addiction^9^.

Altered glutamatergic neurotransmission has been implicated in various phases of the addiction cycle, from initial and voluntary to chronic and compulsory alcohol use^13,14^. This is not of surprise considering that glutamate is the primary excitatory neurotransmitter in the brain^15^. Via its interaction with the mesocorticolimbic dopaminergic system, glutamatergic neurotransmission mediates alcohol-related reward, whereas a hyperglutamatergic state is central to the development of alcohol dependence^16,17^. Key regions of the mesocorticolimbic system are the ventral tegmental area (VTA), which projects to the Acb and the mPFC. Altered glutamatergic transmission is also seen upon both acute and chronic stress in the PFC and midbrain dopamine neurons^18^. Furthermore, ELS has been shown to result in increased excitatory glutamatergic neurotransmission in the paraventricular nucleus of the hypothalamus^19^, and in disturbed homeostasis of glutamatergic synapses^20^, as well as in aberrant reward processing and drug-seeking behaviors, likely mediated by disturbances in dopaminergic and glutamatergic neurotransmission in the PFC and the Acb^21^.

To date, the best markers for the glutamatergic phenotype are the Vesicular Glutamate Transporters (VGLUTs) 1–3 (encoded by the solute carrier superfamily genes *Slc17a7/Vglut1, Slc17a6/Vglut2 and Slc17a8/Vglut3*, respectively), as every neuron that expresses a *Vglut* gene has the ability to release glutamate^22,23^. That is because VGLUTs actively package glutamate into presynaptic vesicles in neurons through an electrochemical proton-dependent gradient^24^. Lately, a number of studies has shown a role of VGLUTs in reward and addiction, including alcohol-related phenotypes in both humans^25–28^ and rodents^29–36^. Altered DNA methylation patterns of the *Vglut2* promoter in the murine hippocampus have been observed as a result of prenatal ethanol exposure^37^. A recent EWAS study found an association between alcohol intake and two CpGs within the body of *VGLUT1* and the promoter of *VGLUT2* in humans^12^. Yet, the interplay between both ELS and adult ethanol consumption on *Vgluts* DNA methylation patterns in the brain of young adults has never been studied.

In fact, the interplay between ELS and ethanol was only recently investigated on *Vgluts* gene expression in young adult outbred Wistar rats^30^. More specifically, previous analysis provided evidence of differential *Vglut1-3* expression in the mesocorticolimbic system [including the VTA, Acb, dorsal striatum (dStr)], and mPFC of adult outbred Wistar rats who had been previously exposed, during the first three postnatal weeks, to ELS as compared to control, as well as to voluntary consumption of ethanol or water during adulthood^30^. Altered expression of *Dnmt1* and *Mecp2* (methyl-CpG-binding protein 2), two key regulatory genes of DNA methylation and the transcription machinery, was also observed as a result of the interaction between ELS and ethanol. The effect was following the same direction as on *Vglut3* in the Acb, but opposite direction than *Vglut1* in the dStr, thus suggesting a potential involvement of the epigenetic machinery in the observed differences of striatal *Vgluts* expression^30^. Alterations in DNA methylation of potential regulatory regions within the promoter or gene body of *Vglut1-3* could be a plausible epigenetic mechanism behind the abovementioned differences in gene expression, and therefore were here studied. The present study sought to investigate the effect of ELS and adult ethanol consumption on *Vglut1-3* DNA methylation levels in the mesocorticolimbic young adult rat brain of relevance to the transcriptional differences previously observed in the same animals. Furthermore, correlations between DNA methylation, and expression, blood corticosterone levels as well as ethanol intake were tested to assess whether they reflect any observed stress or ethanol effects. Finally, moderation effects of DNA methylation on mRNA expression levels were explored.

## Materials and methods

### Animal experiment

The present study is based on the animal experiment presented in^30^ and illustrated in Figure S1. Briefly, adult male Wistar rats (n = 50) were exposed to MS (MS15: control or MS360: ELS) during the first 3 postnatal weeks (PNW), and to water-only [MS15W (n = 10); MS360W (n = 10)] or voluntary alcohol (20%) exposure [MS15E (n = 10); MS360E (n = 20)] during the dark cycle in adulthood (PNW 10–16). Outbred Wistar rats were used to mirror the individual differences and heterogeneity of alcohol-drinking patterns^38^. Only male rats were used to minimize the potential confounding effect of hormonal fluctuations during the estrous cycle in females^39^, as well as based on previous data that alcohol intake in adult female rats was not affected by the MS paradigm^40^. At the end of each session, the ethanol and water intake was quantified by weighing the bottles. The rats were sacrificed at PNW16, immediately after the end of the last two-hour drinking session, and the brain as well as trunk blood for corticosterone analysis were collected and stored at − 80 °C. The study was approved by the Uppsala Animal Ethical Committee (C32/11) and followed the guidelines of the Swedish Legislation on Animal Experimentation (Animal Welfare Act SFS1998:56) and the European Communities Council Directive (86/609/EEC). The study was carried out in compliance with the ARRIVE guidelines.

Blood corticosterone levels (ng ml^−1^) were measured using the ImmuChem Double Antibody Corticosterone 125I RIA kit for rats and mice (MP Biomedicals, Orangeburg, NY, USA), as described in^41^ and are reported in Table S8. DNA/RNA was isolated from the rat VTA, Acb, mPFC and dStr, using AllPrep DNA/RNA/miRNA Universal Kit according to the manufacturer’s protocol (Qiagen AB Sollentuna, Sweden), and quantified using a Nanodrop ND 1000 spectrometer. RNA was converted to cDNA and used to assess the expression of *Slc17a7/Vglut1, Slc17a6/Vglut2, Slc17a8/Vglut3, Dnmt1* and *Mecp2* relative to three housekeeping genes (*Actb, Gapdh and Rpl19*) by real time PCR, as described in^30^. For the present study, the DNA was used for DNA methylation analyses. To minimize potential noise and cost of downstream DNA methylation analyses, for each gene and brain region, samples were selected on the basis of within-group homogeneity in terms of direction of gene expression differences compared to the respective control group [e.g. if MS360E group had significantly higher expression compared to MS15E group, rats within MS360E were assessed individually (by calculating the log2fold difference relative to the mean MS15E expression) to check whether their expression levels were higher (same direction) or lower (opposite direction) than the mean MS15E mRNA levels]. Thus, high within-group heterogeneity led to individual-sample DNA methylation analysis for *Vglut1* and *Vglut2* in the VTA (n = 10/group for MS15W, MS360W, MS15E; n = 18 in MS360E), but pooled-sample DNA methylation analyses for *Vglut1* in the dStr (n = 10/group for MS15W, MS360W, MS15E; n = 17 in MS360E), *Vglut3* in the Acb (n = 10/group for MS15W, MS360W, MS15E; n = 17 in MS360E) and *Vglut2* in the mPFC [n = 10 (MS15W), n = 8 (MS360W), n = 9 (MS15E) and n = 20 (MS360E)], for which homogeneous within-group expression patterns were observed. After the exclusion of the aforementioned samples, the transcriptional levels of *Vglut1-3* were re-tested confirming that the previously reported differences^30^ remained unchanged (data not shown).

### Targeted next generation bisulfite sequencing

**Figure 1:**
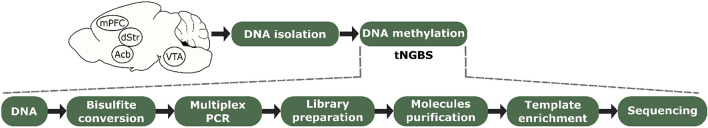
Schematic representation of the DNA methylation analyses using targeted next-generation bisulfite sequencing (tNGBS). *Acb* nucleus accumbens, *dStr* dorsal striatum, *VTA* ventral tegmental area, *mPFC* medial prefrontal cortex.

Targeted next generation bisulfite sequencing (tNGBS) was performed to identify differentially methylated CpG sites in target CpG regions on 48 samples for *Vglut1* and *Vglut2* in the VTA, and 4 pooled samples (one for each experimental group) for *Vglut1* in the dStr, *Vglut3* in the Acb and *Vglut2* in the mPFC. All methylation analyses were performed by EpigenDx Inc (Hopkinton, MA, USA). A flowchart of the procedure is shown in Fig. 1.

*Vglut1-3* gene sequences (+/− 5000 bp including the target array) were acquired from Ensembl genome browser, annotated and converted to a bisulfite sequence using EpigenDx’s Bisulfite Sequence Converter. The target sequences were re-evaluated against UCSC genome browser for repeat sequences and those containing repetitive elements, low sequence complexity, high thymidine content and overall CpG density were excluded for in silico design process. Regions with differential methylation in human gene were retrieved from ENCODE/HAIB and used to design the correspondent rat methylation assays (Figure S2). The 41 designed assays [14 for *Vglut1* (VTA, dStr)*,* 13 for *Vglut2* (VTA, mPFC) and 14 for *Vglut3* (Acb), Table S1] were first grouped by gene, and then by GC %, amplicon size, and design score. A gradient PCR was run on each group of assays at different annealing temperatures using stock bisulfite-modified DNA; the most successful annealing temperature was chosen for sequencing. Assays failing PCR optimization were excluded from analyses.

**Figure 2:**
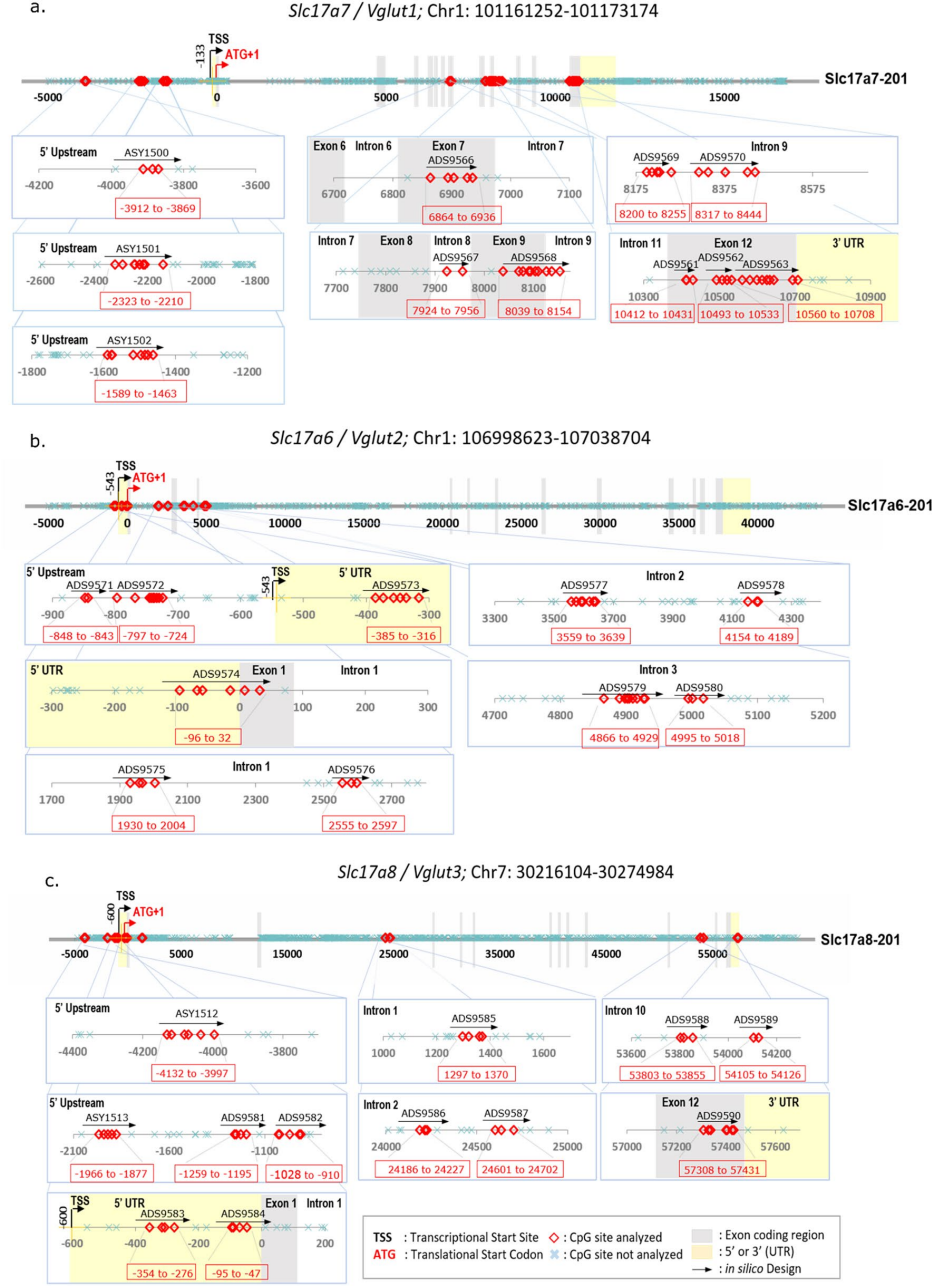
CpGs analyzed within target CpG regions for DNA methylation analysis across the promoter region and gene body of *Slc17a7/Vglut1* (**a**); *Slc17a6/Vglut2* (**b**); *Slc17a8/Vglut3* (**c**). The name of each designed assay is written above the black arrows. In red boxes, the genomic location (in base pairs from ATG) is depicted.

DNA samples (300 ng) were bisulfite modified using Zymo EZ- 96 DNA Methylation Kit (Zymoresearch, CA, USA) as per manufacturer’s protocol with minor modification and were eluted using M-elution buffer in 46 µl. EpigenDx’s custom library preparation method was performed on the chosen test samples, which were then templated using the Ion Chef System (Thermo Fisher, CA, USA) and sequenced using the Ion S5 Sequencer (Thermo Fisher, CA, USA). Read counts from this sequencing run was used to regroup all the assays into final multiplex PCR conditions (Table S1). PCRs included 0.5 units of Qiagen HotStarTaq (Qiagen, MD, Catalogue number 203205), 0.2 µM primers, and 2 µl of bisulfite-treated DNA in a 20 µl reaction. All PCR products were verified and quantified using the QIAxcel Advanced System (Qiagen, Germany). Prior to library preparation, PCR products from the same sample were pooled and purified using QIAquick PCR Purification Kit columns (Qiagen, MD, USA). The final number of successful assays (> 30 reads) was 33 (11 for *Vglut1,* 10 for *Vglut2* and 12 for *Vglut3*). The regions of interest and targeted CpGs for each gene are shown in Fig. 2a–c.

Next, libraries were prepared for all test samples, library molecules were purified using Agencourt AMPure XP beads (Beckman Coulter Inc., CA, USA) and quantified using the QIAxcel Advanced System (Qiagen, Germany). Barcoded samples were then pooled in an equimolar fashion before template preparation and enrichment were performed on the Ion Chef System (Thermo Fisher Scientific Inc., MA, USA) using Ion 520 & Ion 530 ExT Chef reagents. Following this, enriched, template-positive library molecules were then sequenced on the Ion S5 Sequencer using an Ion 530 sequencing chip (Thermo Fisher Scientific Inc., MA, USA).

### Data analysis

FASTQ files from the Ion Torrent S5 server were aligned to the local reference database using open-source Bismark Bisulfite Read Mapper with the Bowtie2 alignment algorithm (https://www.bioinformatics.babraham.ac.uk/projects/bismark/)^42^. Methylation levels were calculated in Bismark by dividing the number of methylated reads by the total number of reads.

### Statistical analysis

The methylation levels of single CpG sites were tested for normality using the Shapiro–Wilk test. The grand majority was not normally distributed, but showed homogeneity of variances (*p* > 0.05), as tested with the modified Levene’s test, based on medians. Thus, interactive effects between ELS and ethanol were assessed using a two-way ANOVA, which is robust to violations of normality^43^, and between-group differences were investigated with the non-parametric Mann–Whitney U test. Correlations between methylation levels and gene expression as well as with corticosterone levels (Table 1) and ethanol intake during the last drinking week (PNW15) before decapitation, and during the last drinking week (PNW16) (Table S10b) were assessed by the non-parametric Spearman co-efficient. PNW15 was chosen for representing better the individual drinking pattern without any extra potential stress effect due to people entering the room to terminate the experiment^30^. Based on PNW15, three subgroups were defined with distinct ethanol intake levels (i.e. high: > 1.5 g/kg/2 h; moderate: 1–1.5 g/kg/2 h and low: < 1 g/kg/2 h drinkers)^30^; Table S10a. Among those subgroups, correlations between methylation and gene expression as well as ethanol intake during PNW15 and PNW16, which is likely more affected by the last ethanol consumption before decapitation, were also assessed (Table S10c).

**Table 1:**
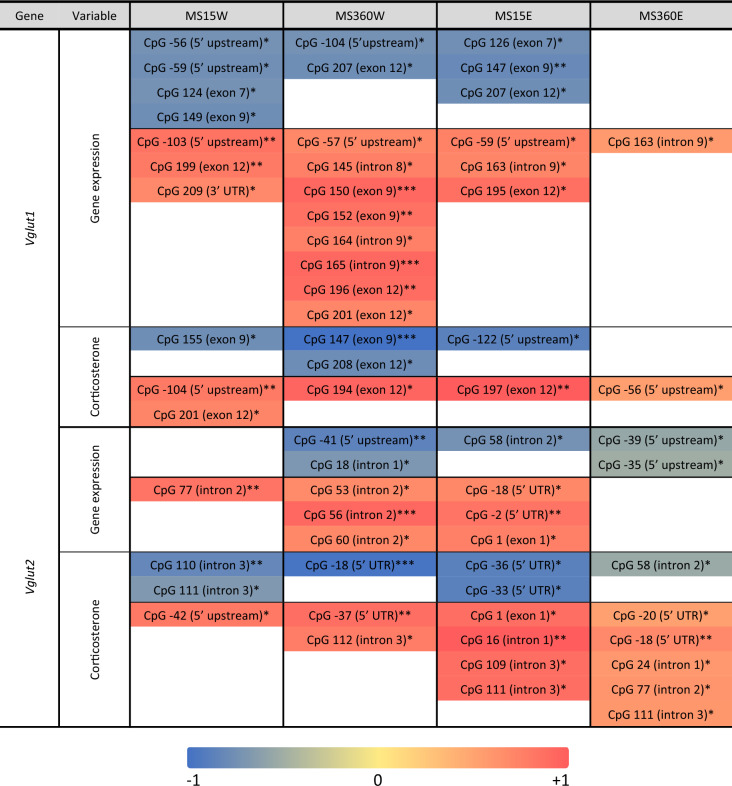
Correlations between CpG methylation and gene expression as well as blood corticosterone levels for *Slc17a7/Vglut1* and *Slc17a6/Vglut2* genes in the VTA by group.

The color bar indicates the Spearman´s *rho* coefficient; * *p* ≤ 0.05; ** *p* ≤ 0.01; *** *p* ≤ 0.001.

Moderated moderation analysis was performed to assess the three-way interaction between rearing [MS15 vs. MS360 (ELS)] and drinking (water vs. ethanol) and *Vglut2* methylation on *Vglut2* expression, using three-way ANOVA and SPSS PROCESS macro v2.16^44^. The Johnson-Neyman technique was used to determine the region of significance in the distribution of methylation levels where the interaction between ELS and ethanol on *Vgluts* expression was significant^44^.

Differences in methylation of each CpG site are reported as absolute percentages (%), and relative (%) differences within brackets. For methylation data of the pooled samples (*Vglut1* in the dStr, *Vglut3* in the Acb and *Vglut2* in the mPFC), for which no statistics could be performed (Table S5-7), CpGs with > 5% difference in absolute values of methylation levels between the groups are reported. Statistical analyses were performed using SPPS (IBM SPSS Statistics for Windows, Version 25.0. Armonk, NY: IBM Corp).

### Transcriptional factor binding sites (TFBS) analysis

**Figure 3:**
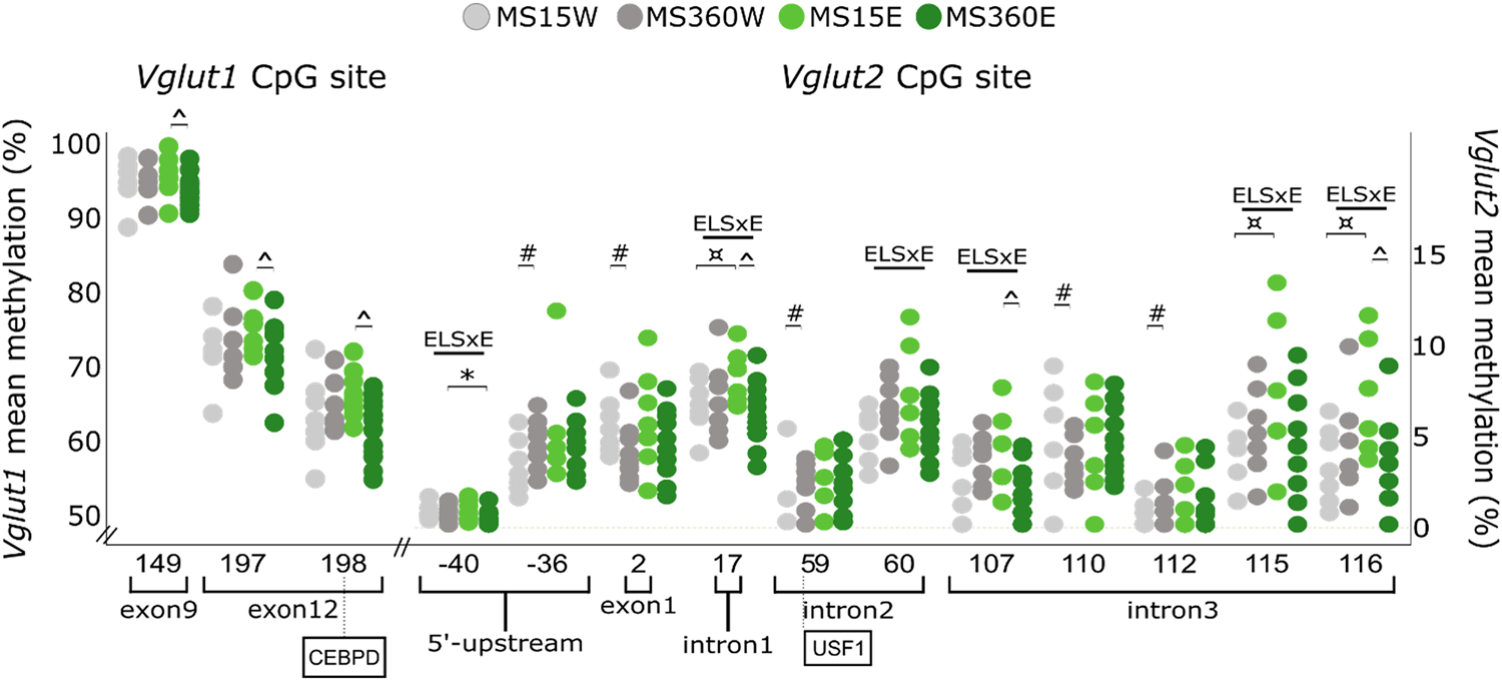
Mean *Vglut1* and *Vglut2* methylation (%) of CpGs associated with interactive effects of ELS and ethanol (ELS x E), tested with two-way ANOVA, and differences between the groups previously found to vary in *Vglut1-2* mRNA levels (i.e. *Vglut1:* ^MS360E, n = 18 vs. M15E, n = 10; *Vglut2*: ^#^MS360W vs. MS15W, n = 10/group) in the ventral tegmental area (VTA), tested with Mann–Whitney U test. In boxes, the transcription factors predicted to bind to the respective site in rat are shown. *CEBPA/D* CCAAT/enhancer binding protein-alpha/delta, *ELS* early life stress, *E* ethanol, *MS* maternal separation, *USF1* Upstream Transcription Factor 1, *UTR* untranslated region, *W* water; Error bars: 95% confidence intervals; * MS360E vs. MS360W; # for MS360W vs. MS15W; ¤ MS15E vs. MS15W; ^ MS360E vs. MS15E; *p* ≤ 0.05. *Vglut1*: Between-group comparisons showed that MS360E rats had lower methylation than MS15E group within exon 9 (CpG149: U = 44; *p* = 0.027) and 12 (CpG197: U = 36; *p* = 0.009; CpG198: U = 33; *p* = 0.005). In silico analysis identified potential binding sites for CCAAT/enhancer binding proteins (C/EBP)-delta at CpG198. *Vglut2:* Interactive effects of ELS and ethanol drinking were seen on methylation of 6 CpGs within the 5’-upstream, and intron 1–3 of *Vglut2.* ELS in ethanol-drinking rats resulted in 0.4–3.7% (19–50%) lower methylation within 5’-upstream (CpG-40), intron 1 (CpG17), 2 (CpG60) and 3 (CpG107, 115, 116), but in 0.25–1.3% (23–55%) higher methylation in no-ELS or water-drinking counterparts (GpG -40: F _(1, 44)_** = **6.526; p = 0.014, partial eta-square = 0.129; adj. R-square = 0.089); CpG17: F _(1, 44)_** = **5.474; p = 0.024, partial eta-square = 0.111; adj. R-square = 0.136; CpG60: F _(1, 44)_** = **6.467; p = 0.015, partial eta-square = 0.128; adj. R-square = 0.076; CpG107: F _(1, 44)_ = 5.175; p = 0.028, partial eta-square = 0.105; adj. R-square = 0.072; CpG115: F _(1, 44)_** = **4.149; p = 0.048, partial eta-square = 0.086; adj. R-square = 0.047; CpG116: F _(1, 44)_ = 10.848; p = 0.002, partial eta-square = 0.197; adj. R-square = 0.251).. Between-group comparisons showed that these effects were driven by lower methylation of CpG-40 (U = 44.5; *p* = 0.027) upon ethanol in ELS-rats (MS360E vs. MS360W) and of CpG17 (U = 32; *p* = 0.004), 107 (U = 48.5; *p* = 0.045), and 116 (U = 28; *p* = 0.002), upon ELS in ethanol-drinking rats (MS360E vs. MS15E), but higher methylation of CpG17 (U = 21; *p* = 0.029), 115 (U = 24; *p* = 0.05), and 116 (U = 11; *p* = 0.002), upon ethanol-only (MS15E vs. MS15W). Between-group comparisons showed that MS360W rats had higher methylation within the 5’-upstream (CpG-36: U = 18; *p* = 0.015), intron 2 (CpG59: U = 18; *p* = 0.015) and 3 (CpG112: U = 21; p = 0.029), but lower methylation within exon 1 (CpG2: U = 22; *p* = 0.035) and intron 3 (CpG110: U = 20; *p* = 0.023) compared to controls (MS15W). In silico analysis identified potential binding sites of Upstream Transcription Factor (USF)-1 at CpG59 (intron 2).

**Figure 4:**
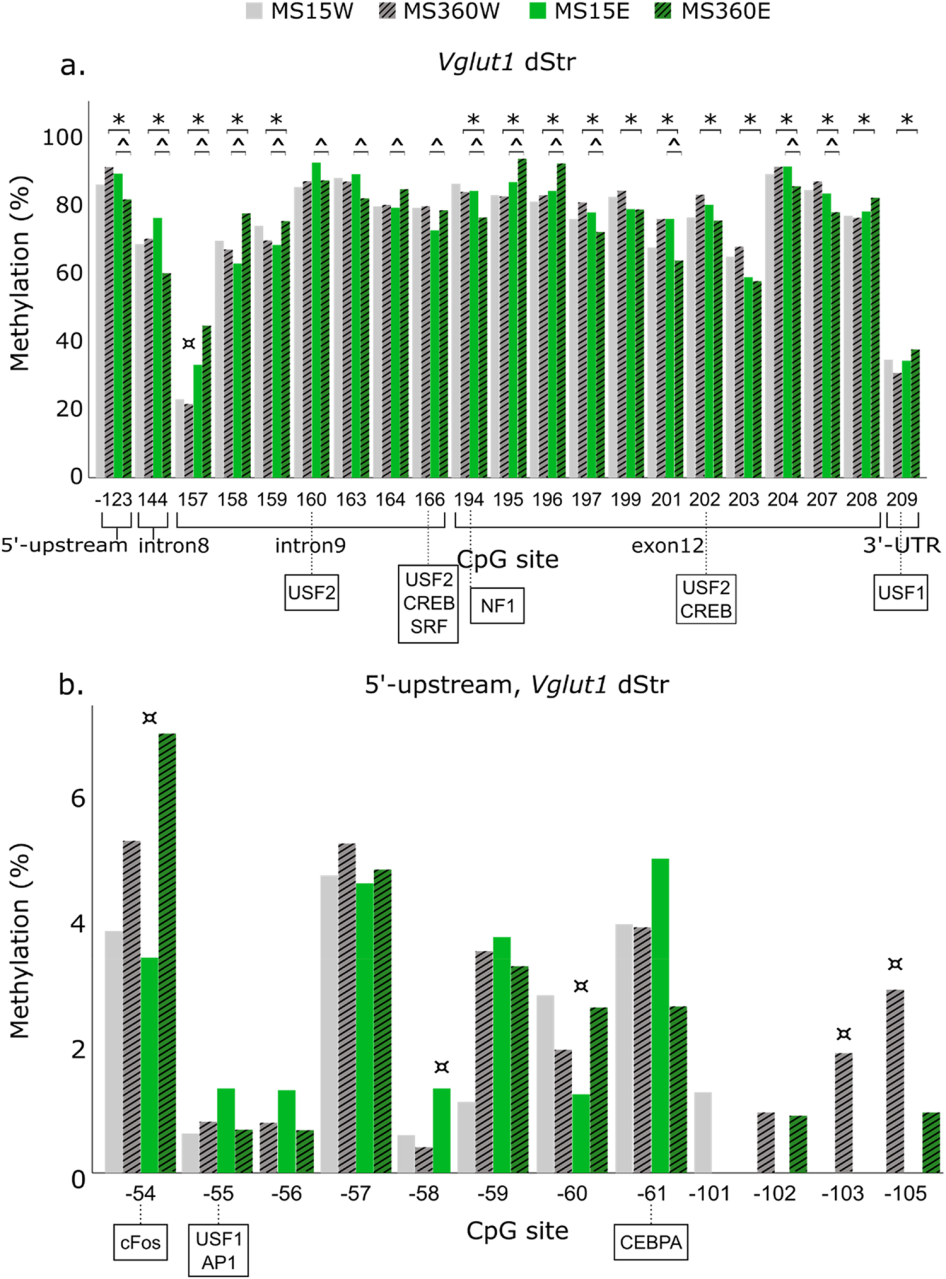
DNA methylation of *Vglut1* CpG sites that differed more than 5% among groups previously found to differ in *Vglut1* expression levels in the dorsal striatum (dStr) (i.e. ^*^ MS360E, n = 17 vs. MS360W, n = 10; ^ MS360E, n = 17 vs. MS15E, n = 10)^30^ (**a**), and within the 5’-upstream in the dStr (**b**). In boxes, the transcription factors predicted to bind to the respective site in rat are shown. *AP1* Activator protein 1, *CEBPA* CCAAT/enhancer binding protein-alpha, *CREB* cAMP Responsive Element-Binding Protein. *E* ethanol, *MS* maternal separation, *NF1* Nuclear factor 1, *SRF* Serum response factor, *USF1-2* Upstream Transcription Factor 1–2, *UTR* untranslated region; *W* water; ¤ > 2 – threefold difference in % methylation when compared to MS360E.

**Figure 5:**
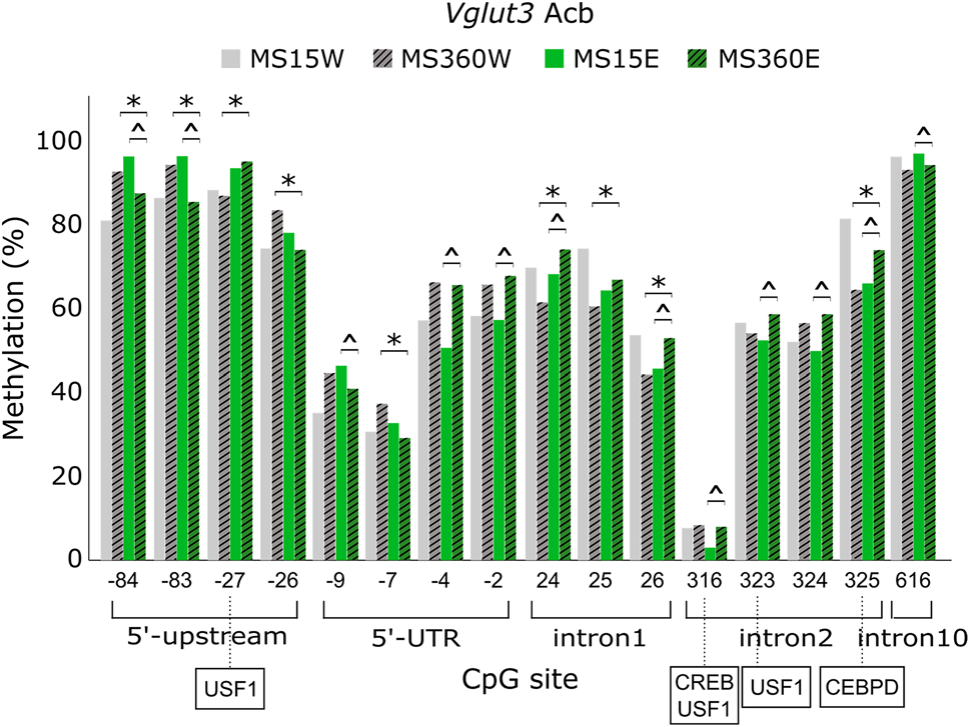
DNA methylation of *Vglut3* CpG sites that differed more than 5% among groups previously found to differ in *Vglut3* expression levels in the nucleus accumbens (Acb) (i.e. *MS360E, n = 17 vs. MS360W, n = 10 and ^MS360E, n = 17 vs. MS15E, n = 10)^30^. In boxes, the transcription factors predicted to bind to the respective site in rat are shown. *CEBPD* CCAAT/enhancer binding protein-delta, *CREB* cAMP Responsive Element-Binding Protein, *E* ethanol, *MS* maternal separation, *USF1* Upstream Transcription Factor 1, *UTR* untranslated region, *W* water.

**Figure 6:**
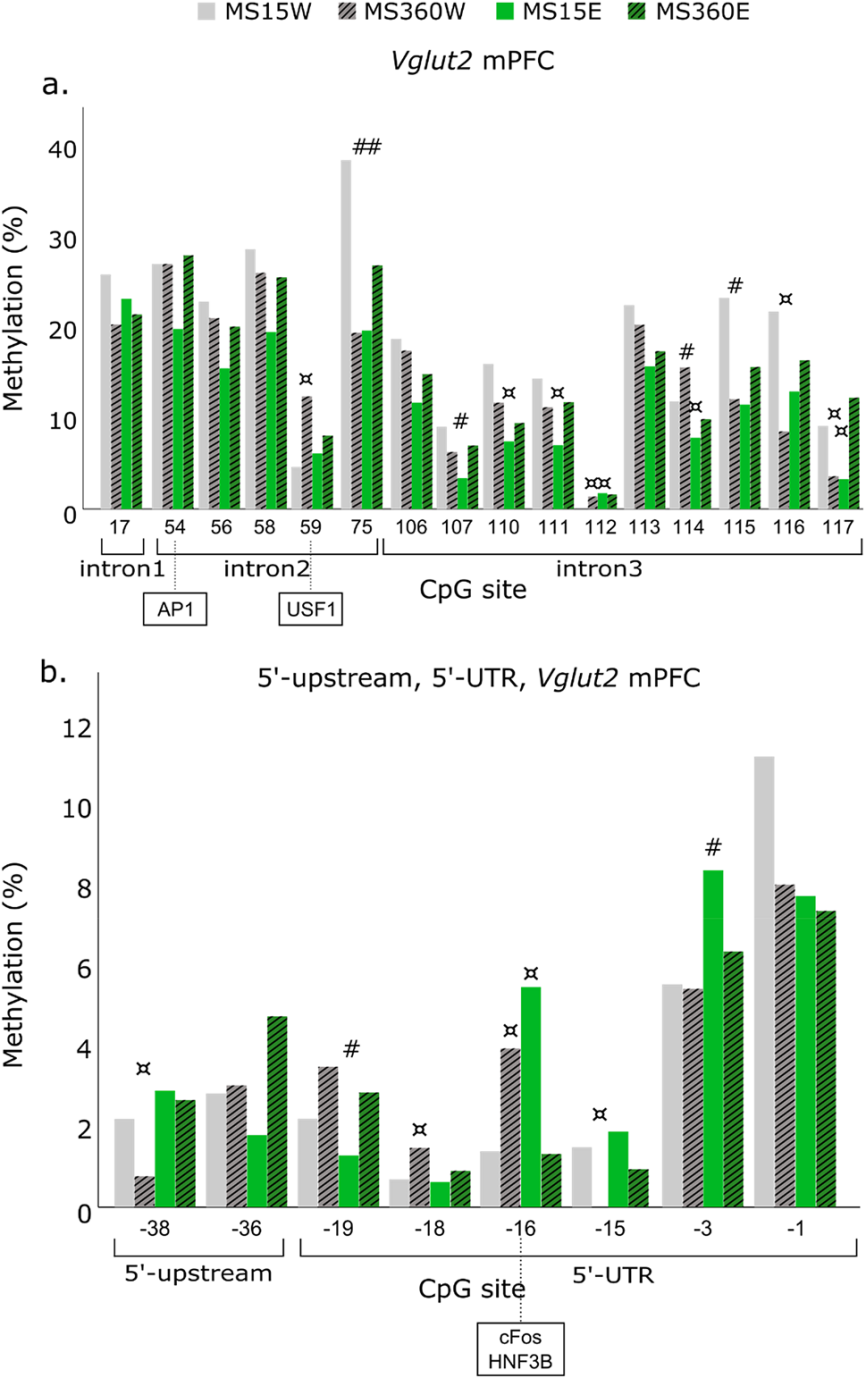
DNA methylation of *Vglut2* CpG sites in the mPFC that differed more than 5% among groups previously found to differ in *Vglut2* expression levels in the medial prefrontal cortex (mPFC) (i.e. MS360W, n = 8 vs. MS15W, n = 10 and MS15E, n = 9 vs. MS15W) ^30^ within intron 1–3 (**a**)**,** and within 5’-upstream and 5’-UTR (**b**). In boxes, the transcription factors predicted to bind to the respective in rat site are shown. *AP1* Activator protein 1, *E* ethanol, *HNF3B* Hepatocyte nuclear factor-3 beta, *MS* maternal separation, *USF1* Upstream Transcription Factor 1, *UTR* untranslated region, *W* water; #: 0.5—onefold and ¤: > 1—threefold difference in % methylation compared MS360W or MS15E to MS15W.

Potential TFBS in rat (*Rattus Norvegicus) Vglut1-3* were assessed using ALGGEN PROMO^45,46^ which predicts TFBSs using TRANSFAC database version 8 (http://alggen.lsi.upc.es/cgi-in/promo_v3/promo/promoinit.cgi?dirDB=TF_8.3) and are depicted in Figs. 3, 4, 5, 6.

## Results

CpGs in potentially regulatory regions of *Vglut1-3* were analyzed using tNGBS. Gene, but not brain region-, specific CpG methylation patterns were observed. CpG-specific methylation differences were noticed between the groups previously found to differ in their *Vglut* expression levels^30^. High correlations (*r* >  ± 0.7) between CpG-methylation and gene expression or corticosterone levels were observed in all groups, but they were sparse and moderate (*r* <  ± 0.6) in the ethanol-drinking ELS-rats (Table 1, S9). Correlations between CpG-methylation and ethanol intake during PNW15 and PNW16 and their directions were similar for the vast majority of CpGs for MS15E, MS360E and MS360E low drinkers, but not for MS360E moderate and high drinkers (Table S10b, c). However, none of the abovementioned differences or correlations survived Bonferonni correction. Nevertheless, considering the exploratory nature of the study, nominally-significant associations are reported.

### CpG methylation patterns in *Vglut1* targeted regions and its associations with ELS and ethanol consumption

Methylation data was available for 61 CpG sites for *Vglut1* (Table S2). In the dStr, methylation analysis failed in MS360E group for 15 CpGs (25%). In the control group (MS15W), CpGs in the promoter region of *Vglut1* were virtually unmethylated (median: 0- 8%) in both the VTA and dStr, except for 3 CpGs with high methylation (65–86%). CpGs in intronic regions were highly methylated (65–88%) as well as in exonic regions (62–95%) with few exceptions with moderate methylation (23–56%) (Table S5). In the VTA (Fig. 3), ELS in ethanol-drinking rats (MS360E vs MS15E) was associated with hypo-methylation of *Vglut1* exon 9 and 12 (3 CpGs). The combination of ELS and ethanol (in MS360E) showed bidirectional effects on *Vglut1* methylation in the dStr (Fig. 4), leading to hypermethylation (> 5%) in a total of 20% of the analyzed CpGs, the majority within intron 9, while 26%, most of them in exon 12, were hypomethylated, compared to both MS360W and MS15E. When considering smaller differences (1–5%) in the 5’-upstream, MS360E had a total of 26% of all analyzed CpGs hypermethylated, while 43% were hypomethylated, compared to both MS360W and MS15E.

### CpG methylation patterns in *Vglut2* targeted regions and its associations with ELS and ethanol consumption

Methylation data was available for 53 CpG sites for *Vglut2* (Table S3). CpGs in the promoter region, 5’-UTR and exon 1 of *Vglut2* were virtually unmethylated (0–11%), while the range in methylation was wider in intronic regions (0–46%) in both the VTA and mPFC (Table S7). In the VTA, ELS in water-drinking rats had a bidirectional effect on *Vglut2* CpG methylation (5 CpGs), while interaction between ELS and ethanol (driven by differences in MS360E vs. MS15E or MS360W) was associated with hypo-methylation of *Vglut2* promoter and gene body (6 CpGs). *Vglut2* CpG methylation did not moderate the previously found interactive effect of ELS and ethanol on *Vglut2* expression^30^. Lastly, ELS- or ethanol-only were largely associated with lower *Vglut2* CpG-specific methylation in the mPFC (Fig. 6).

### CpG methylation patterns in *Vglut3* targeted regions and its associations with ELS and ethanol consumption

Methylation data was available for 51 CpG sites for *Vglut3* (Table S4). Contrary to *Vglut1-2* methylation pattern, *Vglut3* CpGs (Acb) in the promoter region were highly methylated (75–97%) as well as in exon 12 (83–98%), but more moderately in 5’-UTR (31–71%), while the range in methylation was wider in intronic regions (39–97%) with an exception of CpG316 that had low methylation (8%) (Table S6). ELS and ethanol were associated with lower promoter, but higher intronic *Vglut3* CpG methylation in the Acb (Fig. 5).

## Discussion

The present explorative study shows nominally-significant associations between ELS, voluntary ethanol consumption, and CpG-specific *Vglut1-3* methylation in the VTA, Acb, dStr and mPFC of outbred adult male rats, as well as correlations between *Vglut1-2* methylation and expression, blood corticosterone levels and ethanol consumption in the VTA. *Vgluts* methylation varied in a gene- or brain region-dependent way, in the groups previously found to differ in their *Vglut* mRNA expression levels^30^, whereas there was not a pattern of association between these effects and the gene targeted regulatory region (i.e. promoter or gene body). These DNA methylation differences could possibly serve as a mechanism behind the previously reported differences in *Vgluts* expression^30^, via potential changes in binding of distinct transcriptional factors. Even though small-in-magnitude differences are ambiguous in regards to their biological significance^47^, subtle differences (1–10%) in methylation of single CpG sites, such the ones found here, have a potential of functional relevance, not likely as on/off switch of gene expression, but rather in redistribution of the transcriptional landscape, affecting translational isoform production and the proteome^48^. Correlations between *Vgluts* expression and methylation were present in all groups, but were sparse in the ethanol-drinking ELS-rats, suggesting that the molecular mechanisms regulating gene expression and DNA methylation are potentially different in the presence of two aversive environmental factors (ELS and ethanol) as compared to only one or none. Moreover, in the VTA, CpG-specific methylation of both *Vglut1* and *2* was correlated with ethanol intake levels in MS15E and MS360E, but also within the three MS360E subgroups during the last two drinking weeks. Few distinct correlations emerged or others were lost when assessing PNW16 compared to PNW15, but the vast majority of the correlations (or their direction) remained virtually the same between the two weeks in MS15E and MS360E, thus suggesting that the differences in methylation were not due to the acute effect of ethanol.

*Vglut1*: In line with previous findings of higher *Vglut1* expression in the VTA of MS360E rats compared to MS15E^30^, we observed hypomethylation of CpG149 (exon 9) and of CpG197 and 198 (exon 12). A plausible mechanism could involve ELS- and ethanol-induced changes in transcriptional factor (TF) binding. TFs control the levels of gene expression^8^, while methylation in non-CpG islands, and in vicinity (± 100 bp) to the TFBS, could block the TFs binding^49^. Herein, the TF C/EBP-delta was predicted to bind to CpG198. Chronic ethanol consumption has been related with increased C/EBP-delta in the brain^50^, and herein high ethanol intake (in MS360E) during the last week before decapitation was negatively correlated with CpG198 methylation (Table S10). Lower methylation of CpG198, likely due to ethanol drinking in MS360E rats, could potentially lead to increased C/EBP-delta binding contributing to higher *Vglut1* expression. Drug-related epigenetic changes in *Vglut1* have been demonstrated; with cocaine treatment leading to downregulation of *Vglut1* expression as well as hypermethylation of the promoter region of the gene in the Acb of mice^51^, while in a recent EWAS study, one CpG within the gene body of *VGLUT1* was associated with alcohol intake in humans^12^.

In the dStr, higher *Vglut1* expression was also shown in the MS360E compared to both MS15E and MS360W rats^30^. For the latter (MS360E vs. MS360W), lower *Dnmt1* expression was also observed^30^. DNMT1 binds to hemi-methylated sites and maintains DNA methylation signatures during DNA replication^52^. Thus, lower *Dnmt1* expression was hypothesized to contribute to lower methylation of specific *Vglut1* CpGs in the MS360E. Indeed, MS360E had lower methylation of 30% of all analyzed CpGs mainly within 5’-upstream, intron 9 and exon 12*,* compared to MS360W. Compared to both MS360W and MS15E, MS360E rats had a total of 43% of all analyzed CpGs, most of them in exon 12 and 5’-upstream, hypomethylated, while 26%, the majority in intron 9, were hypermethylated. Within the 5’-upstream, smaller differences (1–5%) were in fact observed for the majority of the analyzed CpGs. In the same region, and comparing the same groups (MS360E vs. MS15E and MS360W), small-in-magnitude hypermethylation of CpG − 42 was previously observed in the promoter region of Monoamine Oxidase A (*Maoa*)^53^, along with lower *Maoa* expression^41^. MAOA metabolizes monoamine neurotransmitters such as dopamine and serotonine and has been constantly implicated in behavioral regulation, stress- and alcohol-related phenotypes^54^.

*Vglut2*: Previous analysis showed an interactive ELS x ethanol effect on *Vglut2* expression^30^; driven by ELS rats (MS360W) displaying lower *Vglut2* expression in the VTA, while ethanol had a specular effect leading to higher *Vglut2* expression in ELS rats (MS360E), but lower in no-ELS counterparts (MS15E). Moderation analysis did not provide evidence that CpG methylation moderates the previously observed effect of ELS x ethanol on *Vglut2* expression in the VTA^30^. However, and in line with this interactive effect, 6 CpGs within *Vglut2* promoter and gene body were hypomethylated in MS360E compared to MS15E or MS360W. Our findings corroborate well with the study of Zhang *et. al* that showed adult *Vglut2* up-regulation due to prenatal ethanol exposure, further correlated with decreased DNA methylation of the promoter region in the murine hippocampus of males^37^. Furthermore, the lower *Vglut2* expression in MS360W compared to MS15W rats was accompanied by hypermethylation of CpG-36 (5’-upstream), 59 (intron 2), and 112 (intron 3), but hypomethylation of CpG2 (exon 1), and 110 (intron 3). Lower *Dnmt1* expression in the same animals^30^, may have contributed to the hypomethylation of CpG2, and 110, although no correlations between *Dnmt1* expression and methylation of these sites were observed (Table S9). DNMT3a and 3b, with a role in de novo methylation (Moore et al., 2013), might have also played a role in the present methylation differences, and should be investigated.

Furthermore, lower *Vglut2* expression was found in the mPFC in ELS-only (MS360W) or ethanol-only (MS15E) compared to controls (MS15W)^30^. ELS was associated with hypomethylation of CpG75 (intron 2) and CpG115-117 (intron 3), but hypermethylation of CpG59 (intron 2), similar to the ELS effect on the same CpG in the VTA*.* The TF USF-1 is predicted to bind to CpG59; higher methylation of this site could contribute to reduced binding of USF-1 and in turn to lower *Vglut2* expression observed in both the mPFC and VTA of the same group (MS360W vs. MS15W). In the mPFC, ethanol resulted largely in lower CpG-specific methylation and especially within intron 3. This hypomethylation could potentially have contributed to the lower *Vglut2* expression previously reported^30^, in line with the notion that methylation in gene body is associated with higher gene expression^49^. Ethanol also resulted in lower CpG-specific methylation in the promoter of the stress-related genes *Pomc* (proopiomelanocortin), *Avp* (arginine vasopressin) and *Fkbp5* (FK506 Binding Protein 5) in the pituitary and hypothalamus of the same rats^55^.

*Vglut3:* In the Acb, lower *Vglut3,* and *Dnmt1* expression was previously found in ELS ethanol-exposed rats, whereas the opposite pattern was seen in the water-drinking counterparts^30^. The lower *Dnmt1* was hypothesized to contribute to lower methylation of specific *Vglut3* CpGs in the MS360E group. Indeed, in the Acb of MS360E rats, *Vglut3* methylation was lower in the majority of targeted CpGs within the 5’-upstream, but higher in most of analyzed CpGs within 5’-UTR, intron 1 and 2. Interestingly, CpG316 in intron 2 had more than twofold higher methylation in MS360E compared to MS15E. The TFs USF-2 and CREB were predicted to bind at CpG316. The CREB pathway has been implicated in addiction; especially in the Acb, chronic exposure to various drugs of abuse leads to activation of CREB^56^, while upon chronic alcohol use, CREB has been suggested to modulate connectivity and synaptic plasticity^9^. Herein, the effect of prolonged episodic ethanol consumption was not assessed on CREB itself, but its potential binding to *Vglut3* CpG316 could be blocked by higher methylation in MS360E, contributing to lower accumbal *Vglut3* expression in that group.

Overall, causality could not be assessed in the present study, thus it remains to be investigated whether the abovementioned signatures precede or follow ELS- and/or ethanol- mediated effects on gene expression. Moreover, the potential confounding effect of single housing, which can be an extra social stressor in voluntary ethanol drinking paradigms, on DNA methylation patterns cannot be ruled out. Other epigenetic markers (e.g. histone modifications) that have been associated with ELS^57^ and/or ethanol-related phenotypes^9^ could have been implicated in the previously observed *Vgluts* expression differences, but they were not assessed herein. Nonetheless, DNA methylation is considered one of the principal interfaces between the genome and the environment^48^. Lastly, the present findings should be interpreted with caution considering that bisulfite-treated DNA was analyzed, which although widely used, cannot distinguish between 5-methyl- and 5-hydroxymethylcytosine^58^, limiting our understanding of each marker’s contribution and potential regulatory effect.

To our knowledge, this is the first study to assess the effect of ELS and subsequent voluntary adult ethanol drinking on *Vglut1-3* DNA methylation patterns in the mesocorticolimbic system of outbred adult male rats, and calls for further investigation of the reported effects. Future functional studies on *Vgluts* as well as on downstream effects of ELS and ethanol on VGLUTs protein expression are guaranteed. Determining epigenetic signatures of key-neuronal markers such as VGLUTs, the best markers for the glutamatergic phenotype, in key reward and stress-related brain regions as well as in different stages of ethanol consumption can shed light on the biological underpinnings of alcohol-related phenotypes and AUD.

## Supplementary Information

Supplementary Information.
