# Supplementary material for: DNA methylation of Vesicular Glutamate Transporters in the mesocorticolimbic brain following early-life stress and adult ethanol exposure—an explorative study
Source: Sci Rep. 2021 Jul 28;11:15322. doi: 10.1038/s41598-021-94739-8 (PMC8319394; doi:10.1038/s41598-021-94739-8)
Supplement: Supplementary file 1 — Supplementary Information. [file 41598_2021_94739_MOESM1_ESM.docx]

**DNA methylation of *Vesicular Glutamate Transporters* in the mesocorticolimbic brain following early-life stress and adult ethanol exposure- an explorative study**

Maria Vrettou ^1^, Liying Yan ^2^, Kent W Nilsson ^3, 4^, Åsa Wallén-Mackenzie ^5^, Ingrid Nylander ^6^, Erika Comasco ^1^*

**Supplementary Table 1.** **Multiplex PCR of bisulfite modified DNA samples**

Summary of each Multiple PCR annealing temperature (TA), the amplicon size (bp) per gene and assay, the target loci, total coverage, and PCR bias testing results (shown as Rsquare). Assays in red text failed PCR optimization and thus excluded from further analyses (n.a.: non-assessed)

| **Gene** | **Assay** | **Amplicon Size (bp)** | **TA (°C)** | **No of CpGs** | **Average Reads** | **Average RSquare** |
| --- | --- | --- | --- | --- | --- | --- |
| Slc17a6 | ADS9579 | 145 | 59 | 9 | 140 | 0.885 |
|  | ADS9577 | 150 |  | 8 | 437 | 0.850 |
|  | ADS9575 | 151 |  | 4 | 484 | 0.992 |
|  | ADS9580 | 97 |  | 3 | 107 | 0.972 |
|  | ADS9576 | 110 |  | 3 | 759 | 0.997 |
|  | ADS9571 | 136 |  | 2 | 46 | 0.903 |
|  | ADS9574 | 181 | 56 | 6 | 277 | 0.989 |
|  | ADS9578 | 90 | 50 | 3 | 75 | 0.987 |
|  | ADS9573 | 126 |  | 6 | 153 | 0.991 |
|  | ADS9572 | 134 |  | 9 | 446 | 0.938 |
|  | ADS9564 | n.a | n.a | n.a | n.a | n.a |
|  | ADS9565 | n.a | n.a | n.a | n.a | n.a |
|  | ASY1503 | n.a | n.a | n.a | n.a | n.a |
| Slc17a7 | ADS9570 | 195 | 59 | 5 | 121 | 0.982 |
|  | ADS9567 | 92 |  | 2 | 352 | 0.976 |
|  | ADS9563 | 202 |  | 9 | 227 | 0.973 |
|  | ADS9569 | 128 |  | 5 | 116 | 0.950 |
|  | ASY1502 | 193 |  | 8 | 181 | 0.984 |
|  | ADS9561 | 142 | 59 | 3 | 43 | 0.952 |
|  | ADS9562 | 94 |  | 4 | 280 | 0.975 |
|  | ASY1501 | 175 |  | 7 | 109 | 0.981 |
|  | ASY1500 | 103 |  | 3 | 369 | 0.983 |
|  | ADS9566 | 154 | 59 | 5 | 249 | 0.966 |
|  | ADS9568 | 181 |  | 10 | 174 | 0.952 |
|  | ASY1504 | n.a | n.a | n.a | n.a | n.a |
|  | ASY1505 | n.a | n.a | n.a | n.a | n.a |
|  | ASY1506 | n.a | n.a | n.a | n.a | n.a |
| Slc17a8 | ADS9587 | 198 | 59 | 3 | 59 | 0.985 |
|  | ADS9585 | 124 |  | 4 | 160 | 0.995 |
|  | ADS9582 | 178 |  | 5 | 82 | 0.988 |
|  | ADS9586 | 104 |  | 4 | 487 | 0.986 |
|  | ADS9584 | 99 |  | 4 | 95 | 0.986 |
|  | ADS9589 | 108 | 59 | 2 | 90 | 0.994 |
|  | ADS9581 | 124 |  | 4 | 229 | 0.990 |
|  | ADS9583 | 165 |  | 4 | 79 | 0.980 |
|  | ADS9588 | 154 |  | 3 | 88 | 0.994 |
|  | ASY1512 | 222 |  | 6 | 43 | 0.951 |
|  | ADS9590 | 203 | 50 | 7 | 158 | 0.990 |
|  | ASY1513 | 149 |  | 5 | 3309 | 0.978 |
|  | ASY1514 | n.a | n.a | n.a | n.a | n.a |
|  | ASY1517 | n.a | n.a | n.a | n.a | n.a |

**Supplementary Table 2.** Description of CpG sites (*n* = 61) in *Slc17a7/Vglut1* analyzed using NGBS

| **CpG^a^** | **Rnor 6.0 assembly location** | **Genomic context** | **Base pairs from ATG/TSS** | **Average reads** |
| --- | --- | --- | --- | --- |
| –124 | Chr1:101157473 | 5´ Upstream | -3912/-3779 | 369 |
| –123 | Chr1:101157499 | 5´ Upstream | -3886/-3753 |  |
| –122 | Chr1:101157516 | 5´ Upstream | -3869/-3736 |  |
| –105 | Chr1:101159062 | 5´ Upstream | -2323/-2190 | 109 |
| –104 | Chr1:101159091 | 5´ Upstream | -2294/-2161 |  |
| –103 | Chr1:101159134 | 5´ Upstream | -2251/-2118 |  |
| –102 | Chr1:101159138 | 5´ Upstream | -2247/-2114 |  |
| –101 | Chr1:101159154 | 5´ Upstream | -2231/-2098 |  |
| –100 | Chr1:101159167 | 5´ Upstream | -2218/-2085 |  |
| –99 | Chr1:101159175 | 5´ Upstream | -2210/-2077 |  |
| –61 | Chr1:101159796 | 5´ Upstream | -1589/-1456 | 181 |
| –60 | Chr1:101159807 | 5´ Upstream | -1578/-1445 |  |
| –59 | Chr1:101159809 | 5´ Upstream | -1576/ -1443 |  |
| –58 | Chr1:101159867 | 5´ Upstream | -1518/ -1385 |  |
| –57 | Chr1:101159888 | 5´ Upstream | -1497/ -1364 |  |
| –56 | Chr1:101159899 | 5´ Upstream | -1486/ -1353 |  |
| –55 | Chr1:101159908 | 5´ Upstream | -1477/ -1344 |  |
| –54 | Chr1:101159922 | 5´ Upstream | -1463/-1330 |  |
| +123 | Chr1:101168248 | exon 7 | +6864/+6997 | 249 |
| +124 | Chr1:101168278 | exon 7 | +6894/+7027 |  |
| +125 | Chr1:101168288 | exon 7 | +6904/+7037 |  |
| +126 | Chr1:101168311 | exon 7 | +6927/+7060 |  |
| +127 | Chr1:101168320 | exon 7 | +6936/+7069 |  |
| +144 | Chr1:101169308 | intron 8 | +7924/+8057 | 174 |
| +145 | Chr1:101169340 | intron 8 | +7956/+8089 |  |
| +147 | Chr1:101169423 | exon 9 | +8039/+8172 |  |
| +148 | Chr1:101169454 | exon 9 | +8070/+8203 |  |
| +149 | Chr1:101169463 | exon 9 | +8079/+8212 |  |
| +150 | Chr1:101169475 | exon 9 | +8091/+8224 |  |
| +151 | Chr1:101169478 | exon 9 | +8094/+8227 |  |
| +152 | Chr1:101169488 | exon 9 | +8104/+8237 |  |
| +153 | Chr1:101169494 | exon 9 | +8110/+8243 |  |
| +154 | Chr1:101169510 | exon 9 | +8126/+8259 |  |
| +155 | Chr1:101169521 | exon 9 | +8137/+8270 |  |
| +156 | Chr1:101169538 | exon 9 | +8154/+8287 |  |
| +157 | Chr1:101169584 | Intron 9 | +8200/+8333 | 116 |
| +158 | Chr1:101169595 | Intron 9 | +8211/+8344 |  |
| +159 | Chr1:101169607 | Intron 9 | +8223/+8356 |  |
| +160 | Chr1:101169613 | Intron 9 | +8229/+8362 |  |
| +161 | Chr1:101169639 | Intron 9 | +8255/+8388 |  |
| +162 | Chr1:101169701 | Intron 9 | +8317/+8450 | 121 |
| +163 | Chr1:101169722 | Intron 9 | +8338/+8471 |  |
| +164 | Chr1:101169761 | Intron 9 | +8377/+8510 |  |
| +165 | Chr1:101169811 | Intron 9 | +8427/+8560 |  |
| +166 | Chr1:101169828 | Intron 9 | +8444/+8577 |  |
| +194 | Chr1:101171796 | exon 12 | +10412/+10545 | 43 |
| +195 | Chr1:101171798 | exon 12 | +10414/+10547 |  |
| +196 | Chr1:101171815 | exon 12 | +10431/+10564 |  |
| +197 | Chr1:101171877 | exon 12 | +10493/+10626 | 280 |
| +198 | Chr1:101171892 | exon 12 | +10508/+10641 |  |
| +199 | Chr1:101171904 | exon 12 | +10520/+10653 |  |
| +200 | Chr1:101171917 | exon 12 | +10533/+10666 |  |
| +201 | Chr1:101171944 | exon 12 | +10560/+10693 | 227 |
| +202 | Chr1:101171965 | exon 12 | +10581/+10714 |  |
| +203 | Chr1:101171983 | exon 12 | +10599/+10732 |  |
| +204 | Chr1:101171997 | exon 12 | +10613/+10746 |  |
| +205 | Chr1:101172010 | exon 12 | +10626/+10759 |  |
| +206 | Chr1:101172018 | exon 12 | +10634/+10767 |  |
| +207 | Chr1:101172028 | exon 12 | +10644/+10777 |  |
| +208 | Chr1:101172077 | exon 12 | +10693/+10826 |  |
| +209 | Chr1:101172092 | 3’-UTR | +10708/+10841 |  |

^a^CpG numbers are relative to the ATG transcription start codon, a negative sign indicates location of the CpG site upstream of ATG, and a positive sign indicates location of the CpG site downstream of ATG.

**Supplementary Table 3.** Description of CpG sites (*n* = 53) in *Slc17a6/Vglut2* analyzed using NGBS

| **CpG^a^** | **Rnor 6.0 assembly location** | **Genomic context** | **Base pairs from ATG/TSS** | **Average reads** |
| --- | --- | --- | --- | --- |
| –42 | Chr1:106998318 | 5´ Upstream | -848/-305 | 46 |
| –41 | Chr1:106998323 | 5´ Upstream | -843/-300 |  |
| –40 | Chr1:106998369 | 5´ Upstream | -797/-254 | 446 |
| –39 | Chr1:106998398 | 5´ Upstream | -768/-225 |  |
| –38 | Chr1:106998421 | 5´ Upstream | -745/-202 |  |
| –37 | Chr1:106998424 | 5´ Upstream | -742/-199 |  |
| –36 | Chr1:106998426 | 5´ Upstream | -740/-197 |  |
| –35 | Chr1:106998428 | 5´ Upstream | -738/-195 |  |
| –34 | Chr1:106998432 | 5´ Upstream | -734/-191 |  |
| –33 | Chr1:106998436 | 5´ Upstream | -730/-187 |  |
| –32 | Chr1:106998442 | 5´ Upstream | -724/-181 |  |
| –20 | Chr1:106998781 | 5’-UTR | -385/+159 | 153 |
| –19 | Chr1:106998793 | 5’-UTR | -373/+171 |  |
| –18 | Chr1:106998810 | 5’-UTR | -356/+188 |  |
| –17 | Chr1:106998820 | 5’-UTR | -346/+198 |  |
| –16 | Chr1:106998830 | 5’-UTR | -336/+208 |  |
| –15 | Chr1:106998850 | 5’-UTR | -316/+228 |  |
| –4 | Chr1:106999070 | 5’-UTR | -96/+448 |  |
| –3 | Chr1:106999098 | 5’-UTR | -68/+476 |  |
| –2 | Chr1:106999107 | 5’-UTR | -59/+485 | 277 |
| –1 | Chr1:106999151 | 5’-UTR | -15/+529 |  |
| +1 | Chr1:106999173 | exon 1 | +8/+551 |  |
| +2 | Chr1:106999197 | exon 1 | +32/+575 |  |
| +16 | Chr1:107001095 | intron 1 | 1930/2473 | 484 |
| +17 | Chr1:107001123 | intron 1 | 1958/2501 |  |
| +18 | Chr1:107001131 | intron 1 | 1966/2509 |  |
| +19 | Chr1:107001169 | intron 1 | 2004/2547 |  |
| +23 | Chr1:107001720 | intron 1 | 2555/3098 | 759 |
| +24 | Chr1:107001746 | intron 1 | 2581/3124 |  |
| +25 | Chr1:107001762 | intron 1 | 2597/3140 |  |
| +53 | Chr1:107002724 | intron 2 | 3559/4102 | 437 |
| +54 | Chr1:107002739 | intron 2 | 3574/4117 |  |
| +55 | Chr1:107002757 | intron 2 | 3592/4135 |  |
| +56 | Chr1:107002760 | intron 2 | 3595/4138 |  |
| +57 | Chr1:107002762 | intron 2 | 3597/4140 |  |
| +58 | Chr1:107002785 | intron 2 | 3620/4163 |  |
| +59 | Chr1:107002798 | intron 2 | 3633/4176 |  |
| +60 | Chr1:107002804 | intron 2 | 3639/4182 |  |
| +75 | Chr1:107003319 | intron 2 | 4154/4697 | 75 |
| +76 | Chr1:107003350 | intron 2 | 4185/4728 |  |
| +77 | Chr1:107003354 | intron 2 | 4189/4732 |  |
| +106 | Chr1:107004031 | intron 3 | 4866/5409 | 140 |
| +107 | Chr1:107004055 | intron 3 | 4890/5433 |  |
| +108 | Chr1:107004063 | intron 3 | 4898/5441 |  |
| +109 | Chr1:107004067 | intron 3 | 4902/5445 |  |
| +110 | Chr1:107004070 | intron 3 | 4905/5448 |  |
| +111 | Chr1:107004075 | intron 3 | 4910/5453 |  |
| +112 | Chr1:107004082 | intron 3 | 4917/5460 |  |
| +113 | Chr1:107004092 | intron 3 | 4927/5470 |  |
| +114 | Chr1:107004094 | intron 3 | 4929/5472 |  |
| +115 | Chr1:107004160 | intron 3 | 4995/5538 | 107 |
| +116 | Chr1:107004166 | intron 3 | 5001/5544 |  |
| +117 | Chr1:107004183 | intron 3 | 5018/5561 |  |

^a^CpG numbers are relative to the ATG transcription start codon, a negative sign indicates location of the CpG site upstream of ATG, and a positive sign indicates location of the CpG site downstream of ATG.

**Supplementary Table 4.** Description of CpG sites (*n* = 51) in *Slc17a8/Vglut3* analyzed using NGBS

| **CpG^a^** | **Rnor 6.0 assembly location** | **Genomic context** | **Base pairs from ATG/TSS** | **Average reads** |
| --- | --- | --- | --- | --- |
| –86 | Chr7:30278516 | 5´ Upstream | -4132/-3532 | 43 |
| –85 | Chr7:30278502 | 5´ Upstream | -4118/-3518 |  |
| –84 | Chr7:30278465 | 5´ Upstream | -4081/-3481 |  |
| –83 | Chr7:30278455 | 5´ Upstream | -4071/-3471 |  |
| –82 | Chr7:30278419 | 5´ Upstream | -4035/-3435 |  |
| –81 | Chr7:30278381 | 5´ Upstream | -3997/-3397 |  |
| –47 | Chr7:30276350 | 5´ Upstream | -1966/-1366 |  |
| –46 | Chr7:30276326 | 5´ Upstream | -1942/-1342 |  |
| –45 | Chr7:30276304 | 5´ Upstream | -1920/-1320 | 3309 |
| –44 | Chr7:30276285 | 5´ Upstream | -1901/-1301 |  |
| –43 | Chr7:30276261 | 5´ Upstream | -1877/-1277 |  |
| –33 | Chr7:30275643 | 5´ Upstream | -1258/-659 | 229 |
| –32 | Chr7:30275632 | 5´ Upstream | -1248/-648 |  |
| –31 | Chr7:30275609 | 5´ Upstream | -1225/-625 |  |
| –30 | Chr7:30275579 | 5´ Upstream | -1195/-595 |  |
| –28 | Chr7:30275412 | 5´ Upstream | -1028/-428 | 82 |
| –27 | Chr7:30275405 | 5´ Upstream | -1021/-421 |  |
| –26 | Chr7:30275354 | 5´ Upstream | -970/-370 |  |
| –25 | Chr7:30275302 | 5´ Upstream | -918/-318 |  |
| –24 | Chr7:30275294 | 5´ Upstream | -910/-310 |  |
| –10 | Chr7:30274738 | 5’-UTR | -354/+247 | 79 |
| –9 | Chr7:30274700 | 5’-UTR | -316/+285 |  |
| –8 | Chr7:30274690 | 5’-UTR | -306/+295 |  |
| –7 | Chr7:30274660 | 5’-UTR | -276/+325 |  |
| –4 | Chr7:30274479 | 5’-UTR | -95/+506 | 95 |
| –3 | Chr7:30274474 | 5’-UTR | -90/+511 |  |
| –2 | Chr7:30274455 | 5’-UTR | -71/+530 |  |
| –1 | Chr7:30274431 | 5’-UTR | -47/+554 |  |
| +24 | Chr7:30273088 | intron 1 | +1297/+1897 | 160 |
| +25 | Chr7:30273065 | intron 1 | +1320/+1920 |  |
| +26 | Chr7:30273025 | intron 1 | +1360/+1960 |  |
| +27 | Chr7:30273015 | intron 1 | +1370/+1970 |  |
| +315 | Chr7:30250199 | intron 2 | +24186/+24786 | 487 |
| +316 | Chr7:30250171 | intron 2 | +24214/+24814 |  |
| +317 | Chr7:30250169 | intron 2 | +24216/+24816 |  |
| +318 | Chr7:30250158 | intron 2 | +24227/+24827 |  |
| +323 | Chr7:30249784 | intron 2 | +24601/+25201 |  |
| +324 | Chr7:30249751 | intron 2 | +24634/+25234 | 59 |
| +325 | Chr7:30249683 | intron 2 | +24702/+25302 |  |
| +614 | Chr7:30220582 | intron 10 | +53803/+54403 | 88 |
| +615 | Chr7:30220568 | intron 10 | +53817/+54417 |  |
| +616 | Chr7:30220530 | intron 10 | +53855/+54455 |  |
| +618 | Chr7:30220280 | intron 10 | +54105/+54705 | 90 |
| +619 | Chr7:30220259 | intron 10 | +54126/+54726 |  |
| +641 | Chr7:30217077 | exon 12 | +57308/+57908 | 158 |
| +642 | Chr7:30217056 | exon 12 | +57329/+57929 |  |
| +643 | Chr7:30217047 | exon 12 | +57338/+57938 |  |
| +644 | Chr7:30216985 | exon 12 | +57400/+58000 |  |
| +645 | Chr7:30216980 | exon 12 | +57405/+58005 |  |
| +646 | Chr7:30216960 | exon 12 | +57425/+58025 |  |
| +647 | Chr7:30216954 | exon 12 | +57431/+58031 |  |

^a^CpG numbers are relative to the ATG transcription start codon, a negative sign indicates location of the CpG site upstream of ATG, and a positive sign indicates location of the CpG site downstream of ATG.

**Supplementary Table 5.** Methylation (%) of pooled samples by group of the 61 CpG sites analyzed for *Slc17a7/Vglut1* gene in the dorsal striatum

| median *Vglut1* expression | | | 0,0034144 | 0,0042165 | 0,0031114 | 0,0116529^ |
| --- | --- | --- | --- | --- | --- | --- |
| CpG location  (bp from TSS) | CpG sites | | MS15W | MS360W | MS15E | MS360E |
| 5’-upstream  (-3779 to -1330) |  | -124 | 78,41 | 82,38 | 78 | 80,88 |
|  |  | -123 | 86,49 | 91,65 | 89,67 | 82,14 |
|  |  | -122 | 65,4 | 69,83 | 65,31 | 66,67 |
|  |  | -105 | 0 | 2,91¤ | 0 | ,96 |
|  |  | -104 | 0 | 0 | 0 | 0 |
|  |  | -103 | 0 | 1,90¤ | 0 | 0 |
|  |  | -102 | 0 | ,96 | 0 | ,91 |
|  |  | -101 | 1,28 | 0 | 0 | 0 |
|  |  | -100 | 0 | 0 | 0 | 0 |
|  |  | -99 | 0 | 0 | 0 | 0 |
|  |  | -61 | 3,95 | 3,91 | 5 | 2,65 |
|  |  | -60 | 2,82 | 1,96 | 1,25¤ | 2,63 |
|  |  | -59 | 1,13 | 3,53 | 3,75 | 3,29 |
|  |  | -58 | ,6 | ,41 | 1,34¤ | 0 |
|  |  | -57 | 4,73 | 5,24 | 4,61 | 4,83 |
|  |  | -56 | 0 | ,8 | 1,32 | ,68 |
|  |  | -55 | ,63 | ,82 | 1,34 | ,69 |
|  |  | -54 | 3,85 | 5,28 | 3,42¤ | 6,99 |
| exon 7  (6997-7069) |  | 123 | 80,33 | 78,78 | 76,52 | - |
|  |  | 124 | 86,99 | 81,24 | 83,69 | - |
|  |  | 125 | 87,50 | 92,56 | 87,61 | - |
|  |  | 126 | 93,44 | 93,12 | 96,05 | - |
|  |  | 127 | 94,09 | 92,24 | 91,03 | - |
| intron 8  (8057-8089) |  | 144 | 68,88 | 70,52 | 76,63 | 60,36 |
|  |  | 145 | 56,49 | 55,62 | 49,81 | 52,08 |
| exon 9  (8172-8287) |  | 147 | 91,89 | 93,64 | 98,52 | - |
|  |  | 148 | 91,58 | 95,03 | 93,43 | - |
|  |  | 149 | 93,65 | 91,09 | 100 | - |
|  |  | 150 | 66,67 | 71,58 | 72,59 | - |
|  |  | 151 | 86,56 | 89,70 | 91,85 | - |
|  |  | 152 | 83,61 | 83,91 | 84,96 | - |
|  |  | 153 | 65 | 75,75 | 74,24 | - |
|  |  | 154 | 72,41 | 81,03 | 75,59 | - |
|  |  | 155 | 84,48 | 87,57 | 88,52 | - |
|  |  | 156 | 38,18 | 46,65 | 54,17 | - |
| intron 9  (8333-8587) |  | 157 | 23,13 | 21,79¤ | 33,33 | 44,9 |
|  |  | 158 | 69,84 | 67,33 | 63,16 | 78 |
|  |  | 159 | 74,29 | 70 | 68,67 | 75,73 |
|  |  | 160 | 85,71 | 87,5 | 92,94 | 87,74 |
|  |  | 161 | 84,62 | 81,03 | 79,78 | 80,19 |
|  |  | 162 | 80,85 | 86,52 | 82,35 | 86,49 |
|  |  | 163 | 88,42 | 87,37 | 89,55 | 82,43 |
|  |  | 164 | 80 | 80,46 | 79,66 | 85,14 |
|  |  | 165 | 81,86 | 86,63 | 87,5 | 85,53 |
|  |  | 166 | 79,61 | 80,1 | 73 | 78,91 |
| exon 12  (10545-10826) |  | 194 | 86,67 | 84,31 | 84,62 | 76,79 |
|  |  | 195 | 83,33 | 82,98 | 87,18 | 94,12 |
|  |  | 196 | 81,4 | 83,33 | 84,62 | 92,73 |
|  |  | 197 | 76,32 | 81,27 | 78,28 | 72,55 |
|  |  | 198 | 73,31 | 77,78 | 72,54 | 75,56 |
|  |  | 199 | 82,85 | 84,67 | 79,25 | 79,15 |
|  |  | 200 | 87,91 | 85,55 | 85,08 | 84,5 |
|  |  | 201 | 67,9 | 76,33 | 76,37 | 64,06 |
|  |  | 202 | 76,75 | 83,48 | 80,48 | 75,89 |
|  |  | 203 | 65,2 | 68,19 | 59,15 | 58,04 |
|  |  | 204 | 89,58 | 91,73 | 91,8 | 86,02 |
|  |  | 205 | 77 | 81,62 | 80,95 | 79 |
|  |  | 206 | 92,16 | 92,41 | 94,06 | 93,64 |
|  |  | 207 | 84,82 | 87,41 | 83,84 | 78,35 |
|  |  | 208 | 77,27 | 76,68 | 78,53 | 82,63 |

^: significantly higher gene expression than MS360W and MS15E

¤: > 2 – 3 fold difference in % methylation than MS360E

**Supplementary Table 6.** Methylation (%) of pooled samples by group of the 51 CpG sites analyzed for *Slc17a8/Vglut3* gene in the nucleus accumbens

| median *Vglut3* expression | | | 0,0026349 | 0,0038415 | 0,0035624 | 0,0017039^ |
| --- | --- | --- | --- | --- | --- | --- |
| CpG location  (bp from TSS) | CpG sites | | MS15W | MS360W | MS15E | MS360E |
| 5’-upstream  (-3532 to -310) | -86 | | 94,29 | 90,37 | 92,54 | 88,10 |
|  | -85 | | 97,22 | 94,03 | 93,94 | 93,02 |
|  | -84 | | 81,25 | 93,02 | 96,61 | 87,80 |
|  | -83 | | 86,67 | 94,62 | 96,67 | 85,71 |
|  | -82 | | 93,33 | 92,03 | 95,31 | 95,24 |
|  | -81 | | 97,22 | 93,41 | 94,94 | 93,62 |
|  | -47 | | 93,80 | 93,90 | 93,45 | 93,69 |
|  | -46 | | 94,69 | 95,99 | 95,18 | 95,26 |
|  | -45 | | 93,51 | 95,01 | 92,74 | 94,04 |
|  | -44 | | 92,64 | 92,20 | 93,65 | 91,75 |
|  | -43 | | 94,58 | 94,50 | 94,87 | 95,18 |
|  | -33 | | 91,60 | 92,22 | 90,73 | 91,67 |
|  | -32 | | 89,45 | 90,26 | 89,77 | 89,40 |
|  | -31 | | 93,85 | 92,44 | 92,88 | 92,99 |
|  | -30 | | 88,03 | 88,29 | 87,89 | 85,78 |
|  | -28 | | 95,05 | 90,82 | 93,51 | 88,76 |
|  | -27 | | 88,54 | 87,20 | 93,79 | 95,40 |
|  | -26 | | 74,55 | 83,76 | 78,31 | 74,26 |
|  | -25 | | 87,00 | 85,08 | 89,30 | 87,13 |
|  | -24 | | 88,00 | 86,81 | 83,78 | 88,35 |
| 5’-UTR  (247-554) | -10 | | 33,82 | 38,78 | 36,36 | 34,62 |
|  | -9 | | 35,29 | 44,74 | 46,48 | 41,07 |
|  | -8 | | 36,36 | 52,41 | 45,83 | 50,00 |
|  | -7 | | 30,77 | 37,50 | 32,86 | 29,31 |
|  | -4 | | 57,32 | 66,47 | 50,76 | 65,83 |
|  | -3 | | 71,25 | 76,02 | 72,39 | 77,12 |
|  | -2 | | 58,39 | 65,90 | 57,45 | 68,03 |
|  | -1 | | 68,32 | 63,28 | 61,11 | 59,35 |
| intron 1  (1897-1970) | 24 | | 69,95 | 61,68 | 68,42 | 74,36 |
|  | 25 | | 74,52 | 60,69 | 64,51 | 67,07 |
|  | 26 | | 53,82 | 44,38 | 45,78 | 53,13 |
|  | 27 | | 39,26 | 37,06 | 37,56 | 34,21 |
| intron 2  (24786-25302) | 315 | | 84,94 | 86,30 | 82,29 | 84,86 |
|  | 316 | | 7,73 | 8,41 | 3,02 | 8,02 |
|  | 317 | | 86,59 | 88,03 | 88,66 | 88,75 |
|  | 318 | | 77,45 | 81,48 | 79,89 | 80,94 |
|  | 323 | | 56,76 | 54,23 | 52,55 | 58,82 |
|  | 324 | | 52,17 | 56,70 | 50,00 | 58,82 |
|  | 325 | | 81,69 | 64,67 | 66,19 | 74,19 |
| intron 10  (54403-54726) | 614 | | 92,86 | 91,63 | 92,08 | 92,96 |
|  | 615 | | 91,23 | 96,00 | 93,00 | 95,83 |
|  | 616 | | 96,55 | 93,42 | 97,30 | 94,52 |
|  | 618 | | 94,59 | 90,23 | 89,58 | 93,20 |
|  | 619 | | 91,03 | 87,22 | 84,31 | 91,15 |
| exon 12  (57908-58031) | 641 | | 96,80 | 95,20 | 94,97 | 95,65 |
|  | 642 | | 94,81 | 94,69 | 90,78 | 95,48 |
|  | 643 | | 97,78 | 95,93 | 96,95 | 98,21 |
|  | 644 | | 86,62 | 89,61 | 89,17 | 87,45 |
|  | 645 | | 93,62 | 96,07 | 95,95 | 94,35 |
|  | 646 | | 97,89 | 93,65 | 94,84 | 96,79 |
|  | 647 | | 82,86 | 83,00 | 85,97 | 83,33 |
|  |  | 208 | 77,27 | 76,68 | 78,53 | 82,63 |

^: significantly lower gene expression than MS360W and MS15E

**Supplementary Table 7.** Methylation (%) of pooled samples by group of the 53 CpG sites analyzed for *Slc17a6/Vglut2* gene in the medial prefrontal cortex

| median *Vglut2* expression | | 0,001522 | 0,000801^ | 0,000999^ | 0,001562 |
| --- | --- | --- | --- | --- | --- |
| CpG location  (bp from TSS) | CpG site | MS15W | MS360W | MS15E | MS360E |
| 5’-upstream  (-305 to -181) | -42 | - | 0,00 | 0,00 | 0,00 |
|  | -41 | - | 1,25 | 5,88 | 1,33 |
|  | -40 | 1,25 | 0,91 | 1,35 | 0,44 |
|  | -39 | 0,62 | 0,00 | 0,64 | 0,45 |
|  | -38 | 2,20 | 0,77¤ | 2,90 | 2,67 |
|  | -37 | 1,96 | 1,49 | 1,09 | 0,95 |
|  | -36 | 2,83 | 3,04 | 1,80 | 4,76 |
|  | -35 | 2,90 | 2,02 | 2,61 | 1,82 |
|  | -34 | 1,11 | 1,88 | 1,25 | 1,17 |
|  | -33 | 2,02 | 1,93 | 2,70 | 1,37 |
|  | -32 | 2,76 | 2,32 | 2,47 | 2,77 |
| 5’-UTR  (159-529) | -20 | 3,91 | 3,14 | 2,70 | 2,99 |
|  | -19 | 2,21 | 3,50# | 1,29 | 2,86 |
|  | -18 | 0,69 | 1,48¤ | 0,63 | 0,91 |
|  | -17 | 2,72 | 2,49 | 2,47 | 3,06 |
|  | -16 | 1,39 | 3,96¤ | 5,49¤ | 1,33 |
|  | -15 | 1,49 | 0,00¤ | 1,89 | 0,95 |
|  | -4 | 0,61 | 0,71 | 0,90 | 1,40 |
|  | -3 | 5,56 | 5,45 | 8,40# | 6,38 |
|  | -2 | 3,37 | 2,63 | 2,50 | 3,78 |
|  | -1 | 11,24 | 8,05 | 7,76 | 7,39 |
| exon 1  (551-575) | 1 | 2,96 | 1,90 | 2,73 | 1,39 |
|  | 2 | 5,52 | 5,29 | 4,76 | 7,92 |
| intron 1  (2473-3140) | 16 | 19,45 | 19,84 | 16,24 | 14,76 |
|  | 17 | 25,94 | 20,41 | 23,26 | 21,53 |
|  | 18 | 19,84 | 15,63 | 18,69 | 15,96 |
|  | 19 | 25,92 | 27,63 | 27,32 | 26,38 |
|  | 23 | 43,15 | 43,34 | 44,27 | 41,82 |
|  | 24 | 46,32 | 45,29 | 46,81 | 47,09 |
|  | 25 | 28,33 | 29,43 | 32,19 | 31,40 |
| intron 2  (4102-4732) | 53 | 16,57 | 18,08 | 13,52 | 15,45 |
|  | 54 | 27,09 | 27,11 | 19,91 | 28,05 |
|  | 55 | 29,64 | 32,45 | 25,98 | 28,37 |
|  | 56 | 22,93 | 21,12 | 15,55 | 20,18 |
|  | 57 | 19,23 | 21,82 | 18,04 | 19,75 |
|  | 58 | 28,73 | 26,13 | 19,58 | 25,63 |
|  | 59 | 4,64 | 12,45¤ | 6,16 | 8,12 |
|  | 60 | 21,85 | 21,22 | 17,83 | 18,41 |
|  | 75 | 38,60 | 19,49# | 19,74# | 26,97 |
|  | 76 | 16,67 | 11,86 | 15,58 | 9,30 |
|  | 77 | 12,96 | 9,48 | 9,09 | 8,24 |
| intron 3  (5409-5561) | 106 | 18,80 | 17,52 | 11,76 | 14,90 |
|  | 107 | 9,09 | 6,31 | 3,41# | 7,04 |
|  | 108 | 5,66 | 5,83 | 4,60 | 4,64 |
|  | 109 | 14,56 | 14,93 | 9,83 | 15,51 |
|  | 110 | 16,04 | 11,76 | 7,47¤ | 9,52 |
|  | 111 | 14,42 | 11,26 | 7,06¤ | 11,83 |
|  | 112 | 0,00 | 1,38¤ | 1,75¤ | 1,62 |
|  | 113 | 22,55 | 20,37 | 15,76 | 17,46 |
|  | 114 | 11,88 | 15,67 | 7,88# | 9,95 |
|  | 115 | 23,33 | 12,16# | 11,54¤ | 15,72 |
|  | 116 | 21,84 | 8,57¤ | 13,00 | 16,45 |
|  | 117 | 9,20 | 3,62¤ | 3,30¤ | 12,33 |

^: significantly lower gene expression compared to MS15W

^#^: 0.5 – 1-fold difference in % methylation compared to MS15W

^¤^: > 1 – 3-fold difference in % methylation compared to MS15W

**Supplementary Table 8**. Blood corticosterone levels measured at postnatal week 16 by group

| **Group** | **Corticosterone levels**  (ng/ml) | | |
| --- | --- | --- | --- |
|  | Mean | Min | Max |
| MS15W | 129.9 | 15.6 | 265.3 |
| MS360W | 169.5 | 60.3 | 314.1 |
| MS15E | 142.7 | 55.1 | 276.8 |
| MS360E  Low  Moderate  High | 78.3^  91.7  77  63.4 | 14.6  14.6  46  28 | 195.3  195.3  96.6  91.7 |

^: significantly lower compared to MS360W and MS15E

MS15: maternal separation for 15 min; MS360: maternal separation for 360 min

MS360E: Low drinkers: ethanol intake < 1g/kg/2h; moderate drinkers: ethanol intake 1 – 1.5g/kg/2h; high drinkers: ethanol intake >1.5g/kg/2h

**Supplementary Table 9.** Correlations between *Dnmt1* expression and CpG methylation of *Slc17a7/Vglut1* and *Slc17a6/Vglut2* genes in the VTA by group

| Gene | Variable | MS15W | MS360W | MS15E | MS360E |
| --- | --- | --- | --- | --- | --- |
| *Vglut1* | *Dnmt1 expression* | CpG -56 (5’ upstream)  (*r* = -0.758; *p* = 0.011)  CpG -59 (5’ upstream)  (*r* = -0.697; *p* = 0.025)  CpG 124 (exon 7)  (*r* = -0.721; *p* = 0.019)  CpG 126 (exon 7)  (*r* = -0.661; *p* = 0.038)  CpG 145 (intron 8)  (*r* = -0.818; *p* = 0.004) | CpG -123 (5’ upstream)  (*r* = -0.661; *p* = 0.038) | CpG 164 (intron 9)  (*r* = -0.767; *p* = 0.016) | CpG 150 (exon 9)  (*r* = -0.505; *p* = 0.033)  CpG 152 (exon 9)  (*r* = -0.575; *p* = 0.013) |
|  |  | CpG 154 (exon 9)  (*r* = 0.697; *p* = 0.025)  CpG 162 (intron 9)  (*r* = 0.77; *p* = 0.009)  CpG 165 (intron 9)  (*r* = 0.778; *p* = 0.008)  CpG 198 (exon 12)  (*r* = 0.782; *p* = 0.008)  CpG 199 (exon 12)  (*r* = 0.806; *p* = 0.005) | CpG -102 (5’ upstream)  (*r* = 0.707; *p* = 0.022)  CpG 201 (exon 12)  (*r* = 0.721; *p* = 0.019) | CpG -59 (5’ upstream)  (*r* = 0.8; *p* = 0.01)  CpG 201 (exon 12)  (*r* = 0.767; *p* = 0.016)  CpG 203 (exon 12)  (*r* = 0.7; *p* = 0.036)  CpG 206 (exon 12)  (*r* = 0.75; *p* = 0.02) | CpG -57 (5’ upstream)  (*r* = 0.478; *p* = 0.045)  CpG -61 (5’ upstream)  (*r* = 0.517; *p* = 0.028)  CpG -122 (5’ upstream)  (*r* = 0.544; *p* = 0.02)  CpG 202 (exon 12  (*r* = 0.647; *p* = 0.004)  CpG 203 (exon 12  (*r* = 0.643; *p* = 0.004) |
| *Vglut2* | *Dnmt1 expression* | CpG -19 (5’ upstream)  (*r* = -0.648; *p* = 0.043) |  | CpG -32 (5’ upstream)  (*r* = -0.806; *p* = 0.005  CpG 109 (intron 3)  (*r* = -0.685; *p* = 0.029 | CpG -39 (5’ upstream)  (*r* = -0.489; *p* = 0.039)  CpG 75 (intron 2)  (*r* = -0.579; *p* = 0.012)  CpG 107 (intron 3)  (*r* = -0.65; *p* = 0.003  CpG 108 (intron 3)  (*r* = -0.523; *p* = 0.026 |
|  |  | CpG 77 (intron 2)  (*r* = 0.661; *p* = 0.038) | CpG 60 (intron 2)  (*r* = 0.673; *p* = 0.033) | CpG -40 (5’ upstream)  (*r* = 0.636; *p* = 0.048)  CpG -18 (5’ upstream)  (*r* = 0.709; *p* = 0.022)  CpG 77 (intron 2)  (*r* = 0.648; *p* = 0.043) | CpG 59 (intron 2)  (*r* = 0.552; *p* = 0.018) |

**Supplementary Table 10**

**a.** Ethanol intake during the last week before decapitation [postnatal week (PNW)15] and during the last week (PNW16) with a 2h free choice between water and 20% ethanol

| **Group** | **Ethanol intake** (g/kg/2h) | | | | | |
| --- | --- | --- | --- | --- | --- | --- |
|  | **PNW15** | | | **PNW16** | | |
|  | Median | Min | Max | Median | Min | Max |
| MS15 | 1.32 | 0.39 | 1.77 | 1.32 | 0.5 | 1.84 |
| MS360  Low  Moderate  High | 1.33  0.80  1.33  1.72 | 0.60  0.40  1.04  1.65 | 2.05  0.97  1.45  2.05 | 1.22  0.78  1.16  1.75 | 0.6  0.5  0.58  1.65 | 2.05  1.59  1.84  2.05 |

MS15: maternal separation for 15 min; MS360: maternal separation for 360 min; Low drinkers: ethanol intake < 1g/kg/2h; moderate drinkers: ethanol intake 1 – 1.5g/kg/2h; high drinkers: ethanol intake >1.5g/kg/2h

**b.** Correlations between ethanol intake during the last drinking week before decapitation (PNW15), the last drinking week (PNW16) and CpG methylation of *Slc17a7/Vglut1* and *Slc17a6/Vglut2* genes in the VTA in MS15E and MS360E groups. Highlighted in bold: correlations involving the same CpGs for both weeks

| Gene | Variable | MS15E | | MS360E | |
| --- | --- | --- | --- | --- | --- |
|  |  | PNW15 | PNW16 | PNW15 | PNW16 |
| *Vglut1* | *Ethanol intake* | CpG 150 (exon 9)  (*r* = -0.632; *p* = 0.05)  CpG 161 (intron 9)  (*r* = -0.667; *p* = 0.05)  CpG 197 (exon 12)  (*r* = -0.673; *p* = 0.033)  **Average exon 9**  **(*r* = -0.673; *p* = 0.033)**  Average intron 9  (*r* = -0.733; *p* = 0.016) | CpG -55 (5’ upstream)  (*r* = -0.667; *p* = 0.05)  CpG 155 (exon 9)  (*r* = -0.842; *p* = 0.042)  CpG 155 (exon 9)  (*r* = -0.673; *p* = 0.033)  **Average exon 9**  **(*r* = -0.685; *p* = 0.029)** | **CpG -102 (5’ upstream)**  **(*r* = -0.492; *p* = 0.038)**  CpG -59 (5’ upstream)  (*r* = -0.501; *p* = 0.034)  CpG -55 (5’ upstream)  (*r* = -0.668; *p* = 0.002)  CpG 153 (exon 9)  (*r* = -0.577; *p* = 0.012)  CpG 154 (exon 9)  (*r* = -0.558; *p* = 0.016)  CpG 160 (intron 9)  (*r* = -0.489; *p* = 0.039) | **CpG -102 (5’ upstream)**  **(*r* = -0.556; *p* = 0.016)**  CpG 161 (intron 9)  (*r* = -0.501; *p* = 0.034) |
|  |  | CpG 204 (exon 12)  (*r* = 0.753; *p* = 0.019) |  | CpG 144 (intron 8)  (*r* = 0.542; *p* = 0.02) | CpG 204 (exon 12)  (*r* = 0.736; *p* = 0.001) |
| *Vglut2* | *Ethanol intake* | **CpG 116 (intron 3)**  **(*r* = -0.669; *p* = 0.035)** | CpG -1 (5’ UTR)  (*r* = -0.644; *p* = 0.044)  CpG 18 (intron 1)  (*r* = -0.673; *p* = 0.033)  CpG 108 (intron 3)  (*r* = -0.657; *p* = 0.039)  CpG 114 (intron 3)  (*r* = -0.745; *p* = 0.013)  **CpG 116 (intron 3)**  **(*r* = -0.693; *p* = 0.026)**  Average intron 3  (*r* = -0.673; *p* = 0.033) |  |  |
|  |  | CpG -35 (5’ upstream)  (*r* = 0.758; *p* = 0.011) | CpG -16 (5’ UTR)  (*r* = 0.721; *p* = 0.019)  Average 5’UTR  (*r* = 0.721; *p* = 0.019) |  | CpG 58 (intron 2)  (*r* = 0.653 *p* = 0.003) |

**c.** Correlations between ethanol intake during the last drinking week before decapitation (PNW15), the last drinking week (PNW16) and CpG methylation of *Slc17a7/Vglut1* and *Slc17a6/Vglut2* genes in the VTA in MS360E low, moderate and high drinking groups. Highlighted in bold: correlations involving the same CpGs for both weeks

| Gene | Variable | MS360E_low | | MS360E_moderate | | MS360E_high | |
| --- | --- | --- | --- | --- | --- | --- | --- |
|  |  | PNW15 | PNW16 | PNW15 | PNW16 | PNW15 | PNW16 |
| *Vglut1* | *Ethanol intake* | CpG -58 (5’ upstream)  (*r* = -0.841; *p* = 0.036) | CpG 144 (intron 8)  (*r* = -0.886; *p* = 0.019)  CpG 154 (exon 9)  (*r* = -0.829; *p* = 0.042) | Average exon 12  (*r* = -0.82; *p* = 0.023) | CpG 208 (exon 12)  (*r* = -0.9; *p* = 0.037) | CpG 195 (exon 12)  (*r* = -0.883; *p* = 0.008)  CpG 198 (exon 12)  (*r* = -0.857; *p* = 0.014)  CpG 199 (exon 12)  (*r* = -0.821; *p* = 0.023) |  |
|  |  | **CpG 208 (exon 12)**  **(*r* = 0.829; *p* = 0.042)** | Average 5’upstream (CpG-105 to -99)  (*r* = -0.829; *p* = 0.042)  **CpG 208 (exon 12)**  **(*r* = 0.829; *p* = 0.042** | CpG 126 (exon 7)  (*r* = 0.9; *p* = 0.037)  CpG 197 (exon 12)  (*r* = 0.9; *p* = 0.037) | CpG 153 (exon 9)  (*r* = 0.9; *p* = 0.037)  CpG 158 (intron 9)  (*r* = 0.9; *p* = 0.037)  CpG 163 (intron 9)  (*r* = 0.9; *p* = 0.037)  Average intron 9  (*r* = 0.9; *p* = 0.037) | CpG 150 (exon 9)  (*r* = 0.786; *p* = 0.036)  CpG 165 (intron 9)  (*r* = 0.857; *p* = 0.014) | CpG 145 (exon 9)  (*r* = 0.839; *p* = 0.007) |
| *Vglut2* | *Ethanol intake* | **CpG -36 (5’ upstream)**  **(*r* = -0.943; *p* = 0.005)**  **CpG -20 (5’ upstream)**  **(*r* = -0.829; *p* = 0.042)**  **CpG -19 (5’ upstream)**  **(*r* = -0.886; *p* = 0.019)**  **CpG -18 (5’ upstream)**  **(*r* = -0.943; *p* = 0.005)**  CpG -2 (5’ UTR)  (*r* = -0.886; *p* = 0.019)  **CpG 18 (intron 1)**  **(*r* = -0.829; *p* = 0.042)**  **CpG 19 (intron 1)**  **(*r* = -0.986; *p* = 0.0003)**  CpG 24 (intron 1)  (*r* = -0.943; *p* = 0.005)  **Average 5’UTR**  **(*r* = -0.829; *p* = 0.042)** | **CpG -36 (5’ upstream)**  **(*r* = -0.829; *p* = 0.042)**  **CpG -20 (5’ upstream)**  **(*r* = -0.829; *p* = 0.042)**  **CpG -19 (5’ upstream)**  **(*r* = -0.886; *p* = 0.019)**  **CpG -18 (5’ upstream)**  **(*r* = -0.943; *p* = 0.005)**  **CpG 18 (intron 1)**  **(*r* = -0.886; *p* = 0.019)**  **CpG 19 (intron 1)**  **(*r* = -0.812; *p* = 0.05)**  CpG 25 (intron 1)  (*r* = -0.886; *p* = 0.019)  **Average 5’UTR**  **(*r* = -0.829; *p* = 0.042)** | CpG -37 (5’ upstream)  (*r* = -0.9; *p* = 0.037) | CpG 77 (intron 2)  (*r* = -0.9; *p* = 0.037) | CpG -34 (5’ upstream)  (*r* = -0.821; *p* = 0.023) |  |
|  |  |  |  |  | CpG -38 (5’ upstream)  (*r* = 0.9; *p* = 0.037)  CpG -34 (5’ upstream)  (*r* = 0.9; *p* = 0.037)  CpG -3 (5’ UTR)  (*r* = 0.9; *p* = 0.037) | CpG -20 (5’ upstream)  (*r* = 0.857; *p* = 0.014)  CpG -18 (5’ upstream)  (*r* = 0.964; *p* = 0.0005)  CpG -16 (5’ upstream)  (*r* = 0.786; *p* = 0.036)  CpG -4 (5’ UTR)  (*r* = 0.928; *p* = 0.008) | CpG 109 (intron 3)  (*r* = 0.821; *p* = 0.023)  CpG 114 (intron 3)  (*r* = 0.821; *p* = 0.023) |

In the VTA, among MS360E low, moderate and high drinkers, *Vglut1-2* CpG-specific methylation was correlated [both positively (*r* > 0.8) and negatively (*r <* -0.8)] with *Vglut1-2* expression, as well as ethanol intake during the two last drinking weeks.

Among low drinkers *Vglut1* expression was negatively correlated with methylation of CpG-58 (*r* = -0.986; *p* = 0.00003), 125 (*r* = -0.886; *p* = 0.019), and positively correlated with methylation of CpG203 (*r* = 0.829; *p* = 0.042). Among moderate drinkers, *Vglut1* expression was negatively correlated with methylation of CpG208 (*r* = -0.9; *p* = 0.037), and positively correlated with methylation of CpG153, 158, 163 (for all: *r* = 0.9; *p* = 0.037). Lastly, among high drinkers, *Vglut1* expression was negatively correlated with methylation of CpG-59 (*r* = -0.857; *p* = 0.014), as well as CpG196 and 205 (for both: *r* = -0.786; *p* = 0.036).

Among low drinkers *Vglut2* expression was negatively correlated with methylation of CpG24 and 107 (for both: *r* = -0.829; *p* = 0.042), and positively correlated with methylation of CpG59 (*r* = 0.941; *p* = 0.005). Among moderate drinkers, *Vglut2* expression was negatively correlated with methylation of CpG-39 (*r* = -0.9; *p* = 0.037), and positively correlated with methylation of CpG112 (*r* = 0.894; *p* = 0.041). Lastly, among high drinkers, *Vglut2* expression was negatively correlated with methylation of CpG-42 (*r* = -0.880; *p* = 0.021), and positively correlated with methylation of CpG-15 (*r* = 0.757; *p* = 0.049). *In silico* analysis identified potential binding sites of Hepatocyte Nuclear Factor 3-beta (HNF-3beta) and c-Fos at CpG-16 (5’-UTR) of *Vglut2*.

**Supplementary Figure 1. Animal experiment**

The experimental outline was adapted from (Vrettou et al., 2017). Male Wistar rats were subjected to maternal separation for 15 min (MS15) or 360 min (MS360) during the first three postnatal weeks (PNW). The animals were group-housed during adolescence and single-housed during ethanol/water consumption. The ethanol-drinking rats had free choice between non-sweetened ethanol (5% or 20% made from Etanol 96%; Solveco AB, Rosersberg, Sweden) and water during the dark cycle for three consecutive days a week. The first week (PNW10) the rats had free access to 5% ethanol for 24 h and the next week limited access to 5% for 2 hours; the following five weeks (PNW12 - PNW16) they had access to 20% ethanol in 2-hours sessions for three consecutive days.**
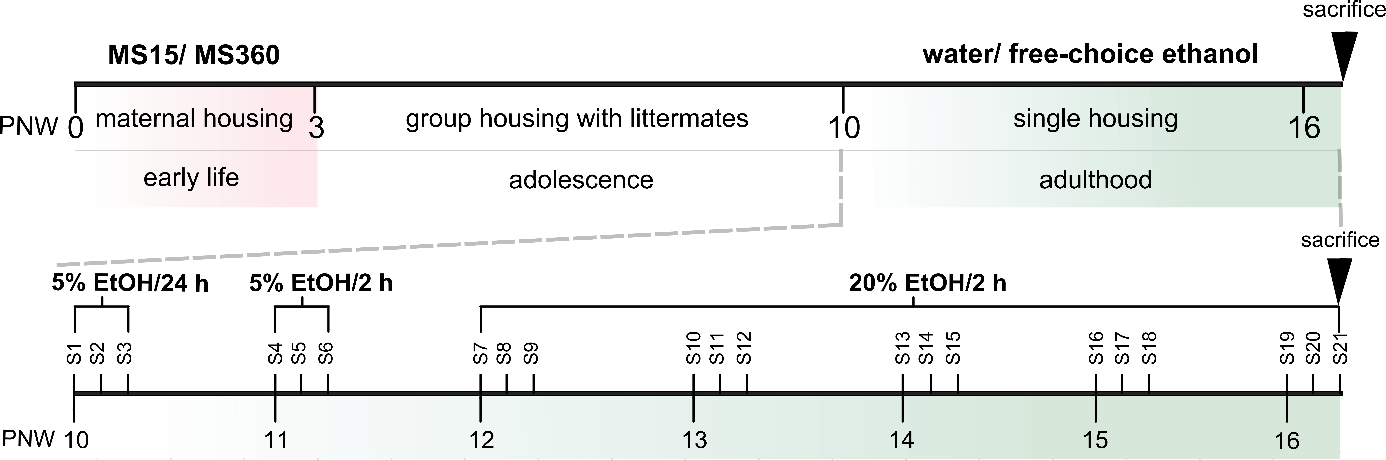
**

EtOH: Ethanol; h: hours; MS15: maternal separation for 15 min; MS360: maternal separation for 360 min; PNW: postnatal week; S: sessions

**Supplementary Figure 2. Gene structure of *SLC17A7/Slc17a7 (VGLUT1/Vglut1)* for human, rat and mouse.**

Gene structures for human, rat, and mouse depicting the CpGs distribution along promoter and gene body for *SLC17A7/Slc17a7 (VGLUT1/Vglut1*). Differential methylated regions in human gene were retrieved from ENCODE/HAIB and used to design the correspondent rat methylation assays.

**
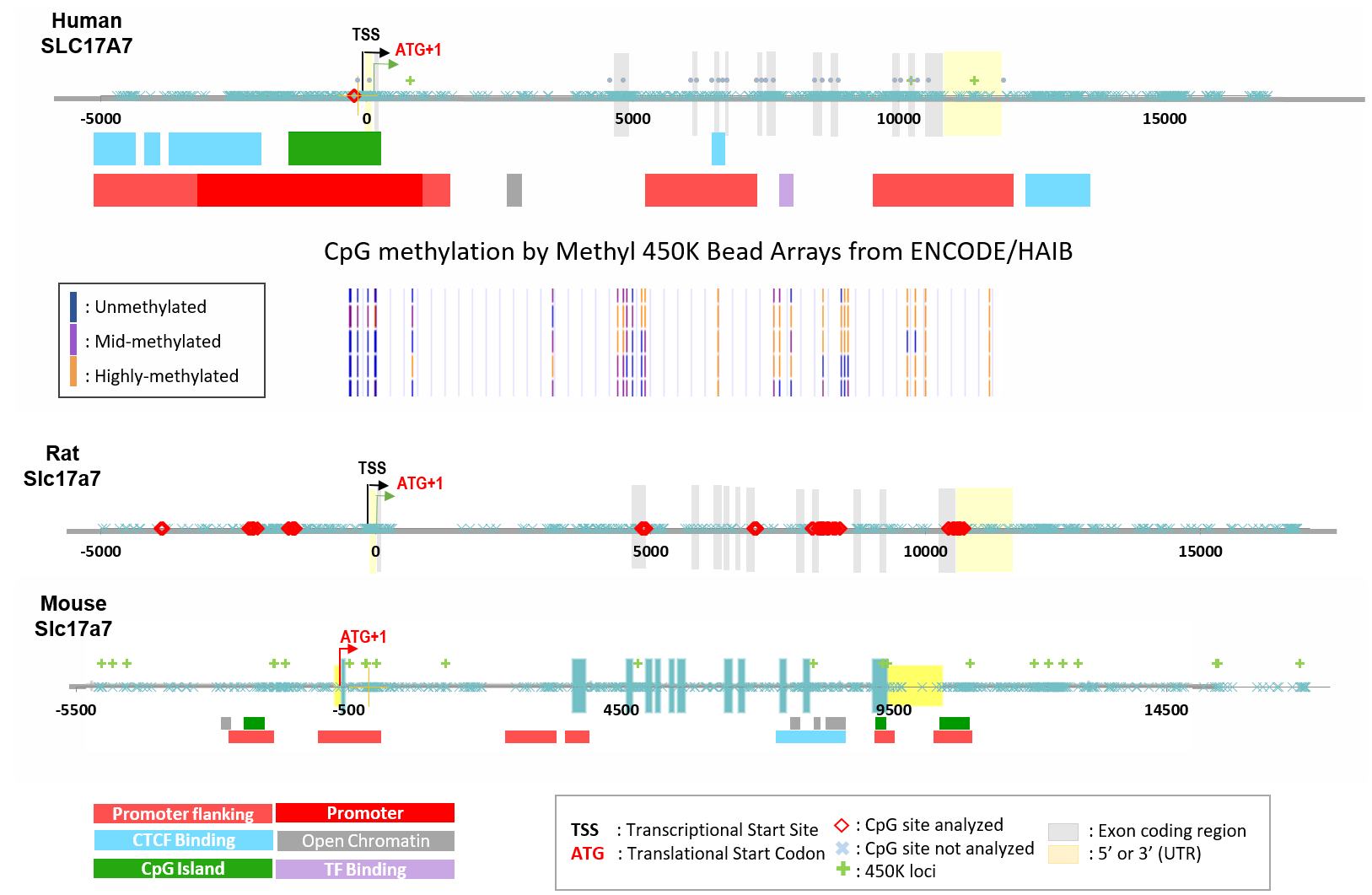
**

VRETTOU, M., GRANHOLM, L., TODKAR, A., NILSSON, K. W., WALLEN-MACKENZIE, A., NYLANDER, I. & COMASCO, E. 2017. Ethanol affects limbic and striatal presynaptic glutamatergic and DNA methylation gene expression in outbred rats exposed to early-life stress. *Addict Biol,* 22**,** 369-380.
